# α-Thio Carbocations (Thionium Ions) as Intermediates in Brønsted Acid-Catalyzed Reactions of Enone-Derived 1,3-Dithianes and 1,3-Dithiolanes

**DOI:** 10.1007/s11244-018-0905-6

**Published:** 2018-03-05

**Authors:** Christoph Brenninger, Thorsten Bach

**Affiliations:** Department Chemie and Catalysis Research Center (CRC), Technische Universität München, Lichtenbergstr. 4, 85747 Garching, Germany

**Keywords:** Acid catalysis, Carbocations, Cyclisation, Dithianes, Photocycloaddition, Sulfonium ions

## Abstract

**Abstract:**

Evidence was collected for the intermediate formation of thionium ions in Brønsted acid-catalyzed [2 + 2] photocycloaddition and electrophilic addition reactions to enone dithianes and dithiolanes. Low-temperature NMR studies helped to elucidate the structure and configuration of the thionium ions and thus support previous and current results obtained by UV/Vis spectroscopy.

**Graphical Abstract:**

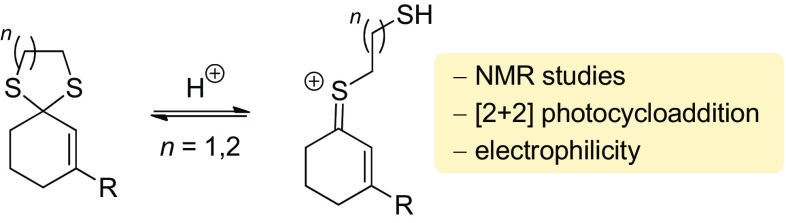

**Electronic supplementary material:**

The online version of this article (10.1007/s11244-018-0905-6) contains supplementary material, which is available to authorized users.

## Introduction

**Fig. 1:**
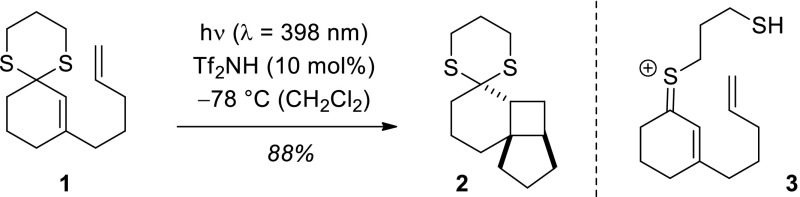
Previously reported Brønsted acid-catalyzed [2 + 2] photocycloadditon of 1,3-dithiane to product **2** and structure of the putative intermediate **3**

The protonation of thiols and thioethers (sulfides) leads to primary and secondary sulfonium ions [1]. Pioneering NMR studies in super acid solution were performed by Olah et al. as early as 1967 [2]. The protonation of 1,3-dithiane was studied by Lambert et al. and a monoprotonated species was detected by ^1^H-NMR and ^13^C-NMR spectroscopy [3]. A second species was identified to which the authors assigned an open chain form with a cationic sulfur atom [4]. A related intermediate called a “carbonium–sulfonium ion” had earlier been observed upon protonation of 1,3-oxathiolane [5, 6]. Similar α-sulfur-substituted carbocations (α-thio carbocations, thionium ions) were invoked in the hydrolysis of *S,O*-acetals [7] and 2-substituted 1,3-dithianes [8]. We became interested in this chemistry when searching for UV/Vis transparent compounds which could be converted by Brønsted acid catalysis into potential chromophores. Based on previous work [9–12] it was conceived that 1,3-dithianes such as **1**, which showed no UV/Vis absorption above λ > 250 nm, would form colored thionium ions which could be photochemically excited at long wavelength. Indeed, the concept turned out to be valid and reactions such as **1** → **2** (Fig. 1) could be promoted with visible light (λ = 398 nm) upon catalysis with strong Brønsted acids, such as HOTf or Tf_2_NH (Tf = trifluoromethanesulfonyl) [13].

The existence of intermediate **3** was supported by its absorption spectrum but was not further substantiated. Its relative configuration has not yet been elucidated. In this paper, we disclose our results on the reaction of a 1,3-dithiolane related to **1** and on UV/Vis experiments with 1,3-dithiolanes and 1,3-dithianes in the presence of Tf_2_NH. NMR spectroscopic evidence for the formation of cation **3** is provided and it was shown that conjugated thionium ions can also undergo a Michael-type addition reaction with a suitable intramolecular nucleophile.

## Experimental

### General Methods

All moisture and air sensitive reactions were carried out in flame-dried glassware under an argon atmosphere using standard Schlenk techniques. Commercially available chemicals were used without further purification. For moisture sensitive reactions tetrahydrofuran (THF) and dichloromethane (CH_2_Cl_2_) were purified using a MBSPS 800 *MBraun* solvent purification system. Dichloromethane for photochemical reactions and Brønsted acid-catalyzed cyclization reactions was additionally dried over activated molecular sieves (4 Å). Dry methanol was obtained from *Acros Organics*. For NMR studies, *Deutero* dichloromethane-*d*_2_ (99.6 at.% D) was employed, which was dried by filtration through a pad of activated basic aluminium oxide under argon atmosphere and was stored over molecular sieves (4 Å).

Photochemical experiments at λ = 366 nm were carried out in Duran tubes (∅ = 1.0 cm) in an RPR-100 photochemical reactor (Southern New England Ultra Violet Company, Branford, CT, USA) equipped with 16 fluorescence lamps (Philips Lighting, Black Light Blue, 8 W, λ = 366 nm). For low temperature irradiation, the reaction vessel was placed in the photoreactor for 20 min prior to irradiation. Flash column chromatography was performed on silica 60 (*Merck*, 230–400 mesh) with the indicated eluent mixture. All solvents for chromatography were distilled prior to use. TLC was performed on silica coated glass plates (*Merck*, silica 60 F254) with detection by UV-light (λ = 254 nm) and/or by staining with a potassium permanganate solution [KMnO_4_] followed by heat treatment.

### Analytical Methods

NMR spectra were recorded at room temperature either on a *Bruker* AVHD-300, AVHD-400, AVHD-500 or an AV-500 cryo. For low temperature measurements (− 70 °C) a *Bruker* DRX400 was used. ^1^H-NMR spectra were referenced to the residual solvent signal of chloroform-d_1_ (CHCl_3_ δ = 7.26 ppm), benzene-d_6_ (C_6_HD_5_ δ = 7.16 ppm) or dichloromethane-d_2_ (CHDCl_2_ δ = 5.32 ppm). ^13^C-NMR spectra were referenced to the ^13^C-D triplet of CDCl_3_ (δ = 77.2 ppm) and C_6_D_6_ (δ = 128.1 ppm), or to the ^13^C-D_2_ quintet of CD_2_Cl_2_ (δ = 54.0 ppm). Apparent multiplets which occur as a result of accidental equality of coupling constants to those of magnetically non-equivalent protons are marked as virtual (*virt*.). The following abbreviations for individual multiplicities were used: *br*-broad, s-singlet, d-doublet, t-triplet, q-quartet, quint.-quintet. Assignments and the multiplicity of the ^13^C-NMR signals were determined by two-dimensional NMR experiments (COSY, HSQC, HMBC, NOESY). Infrared spectra were recorded by the attenuated total reflection (ATR) technique using a *JASCO* IR-4100 spectrometer or a *Perkin Elmer* Frontier IR-FTR spectrometer. The signal intensities are assigned using the following abbreviations: s (strong), m (medium), w (weak). IR signals are reported as wave numbers $\tilde{\upupsilon}$ (cm^−1^). Mass spectra were carried out on a *Agilent* MS5977A MSD spectrometer coupled to a *Agilent* 7890 B gas chromatograph using a HP-5MS UI column (30 m, 0.25 mm, 0.25 µm, 5% diphenyl–95% dimethyl-polysiloxane). HRMS data were determined at a *Thermo Scientific* DFS-HRMS spectrometer. UV/Vis Spectroscopy was performed on a *Perkin Elmer* Lambda 35 UV/Vis spectrometer. Spectra were recorded using a *Hellma* precision cell made of quartz SUPRASIL® with a pathway of 1 mm in dry CH_2_Cl_2_. Concentrations are given for each spectrum.

### Synthetic Protocols

Octahydro-6H-spiro[cyclopenta[1,4]cyclobuta[1,2]-benzene-5,2′-[1,3]dithiolane] (**5**): 7-(Pent-4-en-1-yl)-1,4-dithiaspiro[4.5]dec-6-ene (**4**, 23.4 mg, 97.3 µmol, 1.00 equiv) was dissolved in 9.7 mL CH_2_Cl_2_ (*c* = 10 mM) in a Duran tube and the solution was cooled to − 40 °C. After addition of Tf_2_NH (5.71 mg 19.6 µmol, 0.20 equiv), the solution was irradiated at λ = 366 nm (128 W) for 13.5 h. Irradiation was stopped and NEt_3_ (13.5 µL, 97.3 mmol, 1.00 equiv) was added. The solution was warmed to room temperature and solvent was removed in vacuo. After column chromatography (SiO_2_, P/Et_2_O = 99/1), the title compound was obtained as a colourless oil (18.0 mg, 74.8 µmol, 77%). TLC: *R*_f_ = 0.32 (P/Et_2_O = 99/1) [KMnO_4_]; IR (ATR): $\tilde{\upupsilon}$ (cm^−1^) = 2925 (sp^3^-CH), 2848 (m, sp^3^-CH), 1448 (w, sp^3^-CH), 635 (w, CSC); MS (EI, 70 eV): *m*/*z* (%) = 240 (88) [M]^+^, 212 (99) [M-C_2_H_4_]^+^, 179 (100) [C_11_H_15_S]^+^, 171 (31), 131 (74) [C_5_H_7_S_2_], 91 (41), 79 (34); ^1^H-NMR (500 MHz, CDCl_3_, 298 K): δ (ppm) = 1.19 (*virt*. td, ^2^*J* ≅ ^3^*J* = 12.3 Hz, ^3^*J* = 6.9 Hz, 1H, H-1), 1.37–1.43 (m, 1H, H-8), 1.48–1.53 (m, 1H, H-3), 1.55–1.62 (m, 2H, H-3, H-4), 1.65 (*virt*. td, ^2^*J* ≅ ^3^*J* = 13.0 Hz, ^3^*J* = 6.6 Hz, 1H, H-1), 1.72–1.81 (m, 2H, H-2, H-7), 1.82–1.92 (m, 3H, H-2, H-7, H-8), 1.97 (ddd, ^2^*J* = 12.9 Hz, ^3^*J* = 9.5 Hz, ^3^*J* = 7.5 Hz, 1H, H-4), 2.08–2.14 (m, 1H, H-6), 2.21 (*virt*. tt, ^2^*J* ≅ ^3^*J* = 9.0 Hz, ^3^*J* ≅ 4.9 Hz, 1H, H-3a), 2.33–2.41 (m, 2H, H-4a, H-6), 3.16–3.36 (m, 4H, SC*H*_2_C*H*_2_S); ^13^C-NMR (126 MHz, CDCl_3_, 300 K): δ (ppm) = 20.5 (t, C-7), 25.6 (t, C-2), 27.8 (t, C-4), 29.3 (t, C-8), 32.6 (t, C-3), 34.6 (t, C-6), 38.0 (d, C-3a), 38.1 (t, S*C*H_2_CH_2_S), 39.8 (t, SCH_2_*C*H_2_S), 40.8 (t, C-1), 48.7 (d, C-4a), 49.3 (s, C8a), 70.3 (s, C-5); HRMS (EI, 70 eV): calculated: (C_13_H_20_S_2_): 240.1001; found: 240.1000.

10-Methyl-1,5-dithiadispiro[5.1.5^8^.3^6^]hexadec-10-ene (**12**): 8-(4-Methylpent-4-en-1-yl)-1,5-dithiaspiro[5.5]undec-7-ene (**11**, 23.0 mg, 85.7 µmol, 1.00 equiv) was dissolved in dry CH_2_Cl_2_ (8.6 mL) and the resulting mixture was cooled to − 78 °C. Following the addition of 1,1,2,2,3,3-hexafluoropropane-1,3-disulfonimide (**13**, 1.88 mg 6.43 µmol, 0.10 equiv) the solution was stirred for 5.5 h at − 78 °C in the dark. After which the reaction was quenched via the addition of NEt_3_ (11.9 µL, 84.7 µmol, 1.00 equiv). The solution was allowed to warm to room temperature and the solvent was removed in vacuo. After column chromatography (SiO_2_, P/Et_2_O = 99.5/0.5 → 99/1), the title compound was obtained as a colourless oil (20.1 mg, 73.7 µmol, 87%). TLC: *R*_f_ = 0.35 (P/Et_2_O = 98/2) [KMnO_4_]; IR (ATR): $\tilde{\upupsilon}$ (cm^−1^) = 2926 (s, sp^3^-CH), 1444 (m, sp^3^-CH), 1275 (w), 795 (w, sp^3^-CH); MS (EI, 70 eV): *m*/*z* (%) = 268 (100) [M]^+^, 200 (52) [M-C_5_H_8_]^+^, 159 (60), 118 (62), 105 (58), 91 (32); ^1^H-NMR (500 MHz, CDCl_3_, 298 K): δ (ppm) = 1.28 (ddd, ^2^*J* = 13.1 Hz, ^3^*J* = 9.3 Hz, ^3^*J* = 3.7 Hz, 1H, H-14), 1.36–1.46 (m, 2H, H-13, H14), 1.53–1.58 (m, 1H, H-13), 1.62 (*br* s, 3H, C*H*_3_), 1.60–1.73 (m, 2H, H-15, H-15), 1.78 (d, ^2^*J* = 14.4 Hz, 1H, H-9), 1.82–1.95 (m, 3H, H-3, H-9, H-12), 1.96–2.09 (m, 5H, H-3, H-7, H-7, H-12, H-16), 2.17 (ddd, ^2^*J* = 13.0 Hz, ^3^*J* = 6.9 Hz, ^3^*J* = 3.3 Hz, 1H, H-16), 2.68–2.79 (m, 2H, H-2, H-4), 2.89–2.99 (m, 2H, H-2, H-4), 5.32 (*virt*. tq, ^3^*J* ≅ 3.2 Hz, ^4^*J* = 1.6 Hz, 1H, H-11); ^13^C-NMR (126 MHz, CDCl_3_, 300 K): δ (ppm) = 19.1 (t, C-15), 22.7 (t, C-7), 24.2 (q, *C*H_3_), 25.8 (t, C-3), 26.7 (t, C-2), 26.8 (t, C-4), 33.6 (s, C-8), 35.1 (t, C-13), 36.9 (t, C-14), 38.6 (t, C-16), 42.9 (t, C-12), 47.3 (t, C-9), 49.9 (s, C-6), 119.7 (d, C-11), 132.5 (s, C-10); HRMS (EI, 70 eV): calculated: (C_15_H_24_^32^S_2_): 268.1314; found: 268.1309, calculated: (C_14_^13^CH_24_^32^S_2_): 269.1348; found: 269.1343.

### Low-Temperature NMR Studies

Under an argon atmosphere Tf_2_NH (43.9 mg, 156 µmol, 6.25 equiv) was filled into an NMR tube in a glovebox and was cooled to − 78 °C outside the glovebox. Under argon atmosphere, the acid was dissolved in 0.7 mL dry CD_2_Cl_2_ and 8-methyl-1,5-dithiaspiro[5.5]undec-7-ene (**9**, 5.00 mg, 25.0 µmol, 1.00 equiv) was added. After mixing, the orange-yellow solution was warmed to 203 K (− 70 °C) in a *Bruker* DRX400 spectrometer and spectra were recorded at 203 K. For ^13^C-NMR and 2D experiments dithiane **9** (20.0 mg, 100 µmol, 1.00 equiv) and Tf_2_NH (175 mg, 624 µmol, 6.25 equiv) were dissolved in 0.6 mL dry CD_2_Cl_2_.

## Results and Discussion

### Photochemistry

**Fig. 2:**
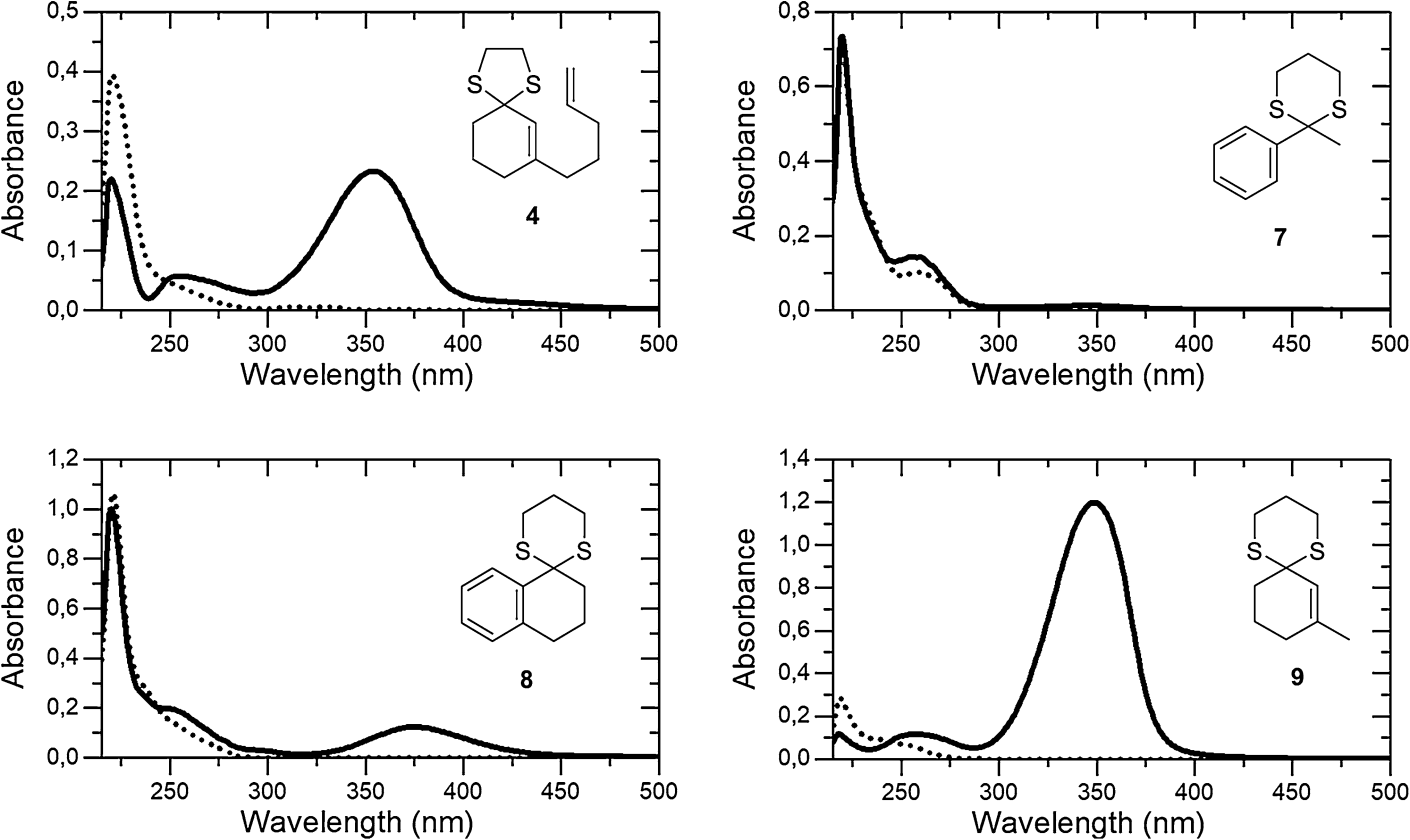
UV/Vis absorption spectra of compounds **4, 7**–**9** in CH_2_Cl_2_ solution without (·····) and with added Brønsted acid (Tf_2_NH, see narrative)

Attempts to induce an intramolecular [2 + 2] photocycloaddition of 1,3-dithiolane **4** were unsuccessful if the irradiation was performed at λ = 398 nm. Gratifyingly, we found that the reaction was feasible once a short-wavelength light source was employed. At λ = 366 nm, a complete conversion to the desired cyclobutane **5** was observed after six hours in the presence of either 10 or one equivalent (equiv) of Brønsted acid (Table 1, entries 1, 2). At a lower catalyst loading of only 0.1 equiv the reactions remained incomplete and starting material could be recovered (entries 3, 4). A good compromise was a catalyst loading of 20 mol% (0.2 equiv) which allowed for full conversion after 13.5 h and resulted in a yield of 77%. The reason for the diminished reactivity of dithiolane **4** was found to be its somewhat lower basicity and/or lower tendency to form a thionium ion. If treated with the Brønsted acid Tf_2_NH the UV/Vis absorption (*c* = 0.5 mM in CH_2_Cl_2_ solution) did not reach saturation even upon addition of 20 equivalents of the acid (Fig. 2). The absorbance at λ = 353 nm for putative intermediate **6** was only 0.2335 (path length of the cuvette *l* = 0.1 cm). In stark contrast, thionium ion **3** had shown saturation under otherwise identical condition already with 12.5 equiv of Tf_2_NH and had produced an absorbance of 1.195 [13]. At λ = 356 nm a molar absorption coefficient of ε = 23,900 M^−1^ cm^−1^ was thus calculated according to the Lambert–Beer law.

**Table 1:** Intermolecular [2 + 2] photocycloadditon of 1,3-dithiolane **4** to product **5** via putative intermediate **6**

Entry^a^	Tf_2_NH (equiv)	*t* (h)	Yield (4) (%)	Yield (5) (%)
1	10	6	–^b^	79
2	1.0	6	–^b^	92
3	0.1	10	27	49
4	0.1	20	14	71
5	0.2	13.5	–^b^	77

^a^All reactions were performed on a scale of 0.1 mmol (*c* = 10 mM) with 16 fluorescence lamps as light sources

^b^The reaction went to full completion and no starting material could be re-isolated

### UV/Vis Spectra

We further explored the structural parameters which favor the formation of thionium ions. While Satchell and co-workers had already observed colored intermediates if treating 2,2-diaryl-1,3-dithianes with perchloric acid [12], the 1,3-dithiane **7** (*c* = 1.0 mM) derived from acetophenone could not be converted into a species with a more intense chromophore (20 equiv Tf_2_NH) and it is thus likely that there is no thionium ion being formed or that its concentration is very low at best. The more rigid 1,3-dithiane **8** (*c* = 1.0 mM) derived from α-tetralone exhibited a detectable absorption upon acid treatment (20 equiv Tf_2_NH) although the measured absorbance was not very high (A = 0.1238). Still, it is evident that the rigid ring system facilitates formation of the respective thionium ion by placing the conjugated arene π-system in proper conjugation with the cationic π-system of the cation. Similarly, the 1,3-dithiane **9** (*c* = 0.5 mM) derived from 2-methyl-2-cyclohexenone is stabilized by the olefinic π-system and the formation of a thionium ion seems to be facile. Indeed, the compound behaved similar to dithiane **1** and produced quantitatively the respective cation upon addition of 12.5 equiv of Tf_2_NH. The calculated absorption coefficient at λ = 349 nm was ε = 23,940 M^−1^ cm^−1^. By comparison with the 1,3-dithiane derived from 2-pentyl-2-cyclohexenone (see Supporting Information) it was shown that the slightly hypsochromic absorption shift as compared with **1** (vide supra) is due to the length of the different alkyl chain but not due to the absence of the olefinic double bond.

### Low-Temperature NMR Studies

**Fig. 3:**
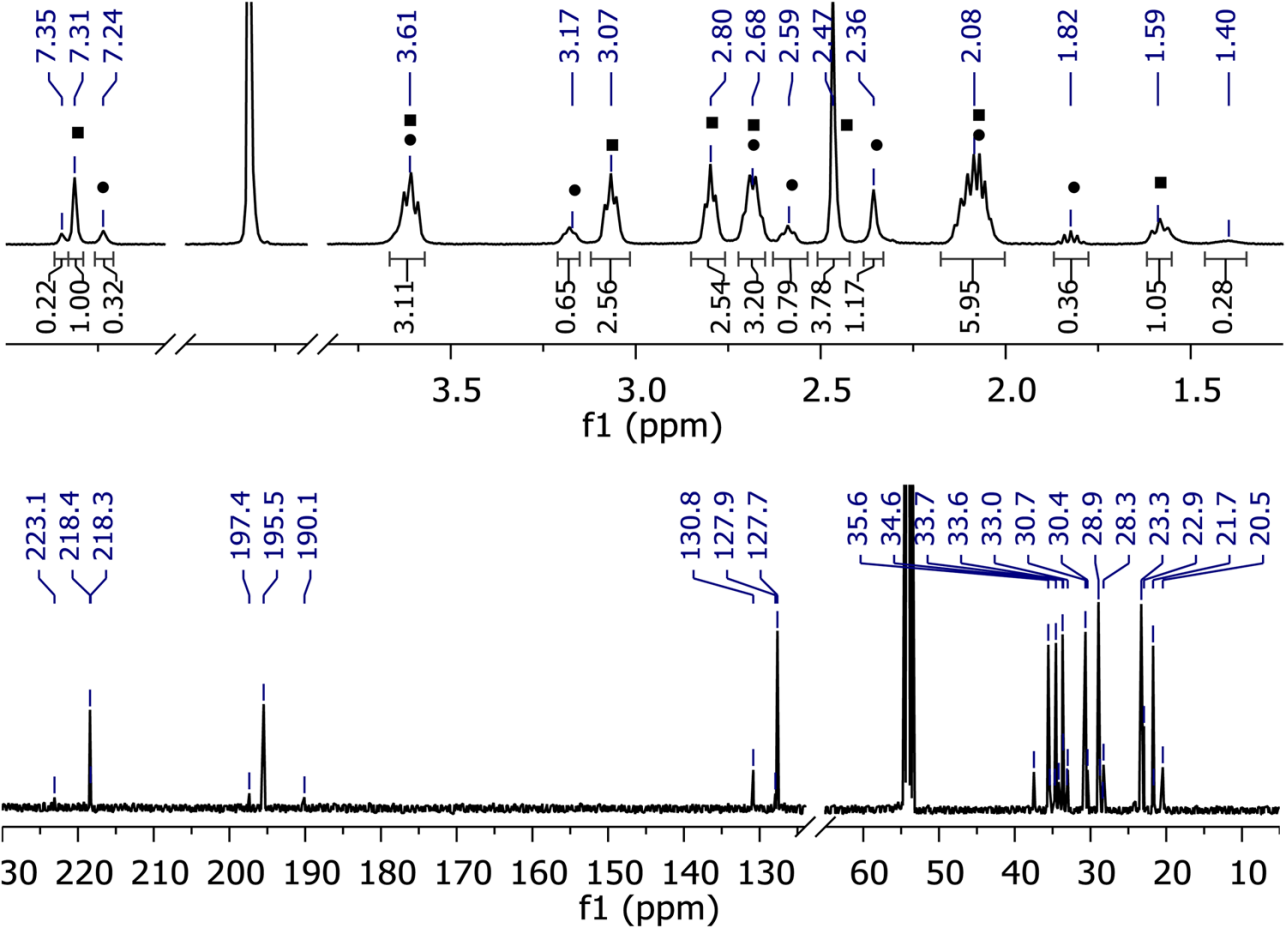
^1^H- and ^13^C-NMR spectra of compound **9** in CD_2_Cl_2_ solution at − 70 °C upon addition of 6.25 equivalents of Tf_2_NH

Given the apparently clean formation of a thionium ion from **9** and given the less complex structure of **9** compared to **1**, it was attempted to substantiate the formation of the cation by low-temperature NMR studies. Upon addition of 6.25 equivalents of Tf_2_NH at − 70 °C, a solution of compound **9** in CD_2_Cl_2_ produced a ^1^H-NMR spectrum (Fig. 3) in which there were no signals of compound **9** detectable. Rather two new species had formed in a ratio of about 3/1. The respective signals are marked as ▪ (major) and ● (minor) in the spectrum. A third species was identified based on its signals at high (δ = 1.40 ppm) and low field (δ = 7.35 ppm). All other signals of this species overlap with the signals of the major isomer and they cannot be distinguished at higher concentration which are necessary for a sufficient signal-to-noise ratio in 2D NMR experiments. Upon addition of acid, the structure of 1,3-dithiane **9** changes, indicated by the fact that the two enantiotopic hydrogen atoms in 5-position of the 1,3-dithiane ring are no longer split into two signals (δ = 1.68 and 1.91 ppm). Rather they appear in a single broad peak centered at δ = 2.08 ppm overlapping with signals from other methylene protons.

**Fig. 4:**
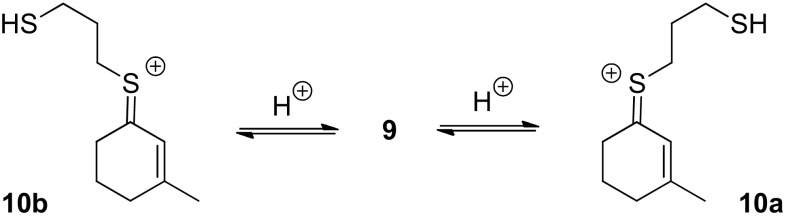
Formation of thionium ions **10** as mixture of two diastereoisomers **10a** (^1^H-NMR signals in Fig. 3: filled square) and **10b** (^1^H-NMR signals in Fig. 3: filled circle)

The resonance of the olefinic hydrogen atom of **9** is shifted by 1.92–2.03 ppm from δ = 5.32 ppm to δ = 7.35, 7.31, and 7.24 ppm for each species. The strong downfield shift can be explained by an electron withdrawing atom or group, generated by the acid. The chemical shift at δ = 3.61 ppm is attributed to a methylene group next to a thionium ion, as already observed by Lambert et al. [3]. In the upfield region, three signals of possible HS- groups (δ = 1.82, 1.58, and 1.40 ppm) are visible indicating a ring opening of the dithiane. The signals in the ^13^C-NMR spectrum at δ = 23.3 and 20.5 ppm also indicate the presence of a thiol bound to an alkyl chain [14]. The ^13^C-NMR spectrum further exhibits downfield shifted signals between 190.1 and 197.4 ppm which are assigned to the olefinic quartenary carbon atoms in conjugation to the carbocation center. The cation center itself resonates at 218.3–223.1 ppm in the three species mentioned above. The ^13^C-NMR signals of the sp^2^ hybridized carbon atoms of all three species appear in three groups of three signals each (δ = 218.3–223.1, 190.1–197.4 and 127.7–130.8 ppm), suggesting a structural similarity of all three species. In a NOESY experiment cross peaks between the signals of the methyl groups (δ = 2.47 and 2.36 ppm), the olefinic hydrogen atoms (δ = 7.31 and 7.24 ppm) and the endocyclic methylene group in β position to the positive charged sulfur atom (δ = 3.07 and 3.17 ppm) were observed for the major and minor species.^1^ The cross peaks are in phase with the diagonal resonance due to chemical exchange by interconversion of both species into one another on the ^1^H-NMR timescale [15]. A chemical exchange is also indicated between two signals of the major species (δ = 3.61 and 2.68 ppm), which can be assigned to the exocyclic methylene groups next to the sulfur atoms. Additionally, the olefinic hydrogen atom of the major species (δ = 7.31 ppm) shows cross peaks with its methyl group (δ = 2.47 ppm) and with both exocyclic methylene groups next to the sulfur atoms (δ = 3.07 and 2.68 ppm), which are in opposite phase with the diagonal peaks due to an intramolecular NOE. The latter NOE indicates a spatial proximity of the olefinic hydrogen atom and the exocyclic methylene groups. In the minor species, the resonances of the methyl (δ = 2.47 ppm) group and the olefinic hydrogen atom (δ = 7.24 ppm) are shifted upfield (Δ δ = −0.07 and − 0.09 ppm) compared to the major species. Additionally, the signal of the endocyclic methylene group in β position to the positive charged sulfur atom (δ = 3.17 ppm) is shifted downfield (Δ δ = + 0.07 ppm) compared to the major species. This observation suggests that the major and the minor species are configurational isomers. Based on the NMR data, structure **10a** was assigned to the major (*Z*)-diastereoisomer of the thionium ions and structure **10b** was assigned to the (*E*)-diastereoisomer (Fig. 4). The structure of the minor component remains unclear. It is conceivable that the intermediate is a rotamer of compound **10a** as its NMR data are very similar to **10a**.

### Thermal Acid-Catalyzed Cyclization

**Fig. 5:**
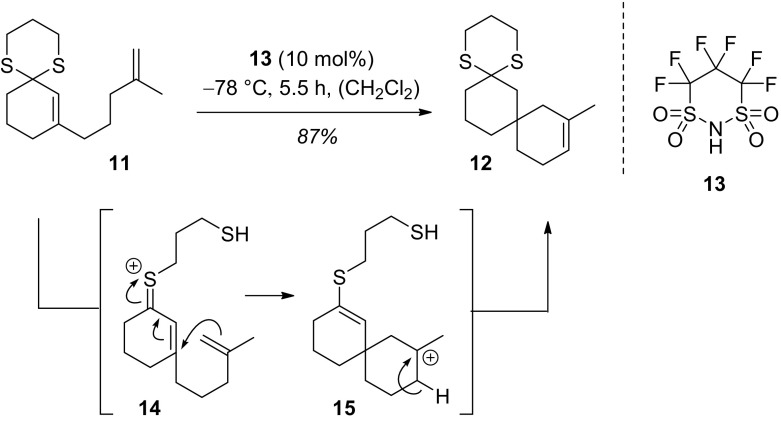
Brønsted acid-catalyzed cyclization of enone 1,3-dithiane **11** to spiro compound **12** via putative intermediates **14** and **15**

In order to collect further evidence for a cationic intermediate formed from enone 1,3-dithianes, we attempted to trap the thionium ion by a suitable nucleophile (Fig. 5). Indeed, when compound **11** was treated with acid **13** [16, 17] there was a reaction in the absence of light and product **12** was isolated in high yield [18, 19]. Cation **14**^2^ seems to be sufficiently electrophilic to attack the nucleophilic 1,1-disubstituted alkene intramolecularly with concomitant formation of tertiary carbocation **15**. Product formation occurs subsequently upon proton elimination and re-cyclization of the thiol to the 1,3-dithiane.

## Conclusion

In summary, it was shown that thionium ions are useful transient intermediates which can be generated upon treatment of enone 1,3-dithianes or 1,3-dithiolanes with a suitable Brønsted acid. The most characteristic feature of these ions is their strong absorption at long wavelength which allows for photochemical reactions to be performed under acidic catalysis. Apart from [2 + 2] photocycloaddition reactions other transformations seem feasible and research along these lines is ongoing in our laboratories.

## Electronic supplementary material

Below is the link to the electronic supplementary material.

Supplementary material 1 (PDF 1740 KB)

## Footnotes

1 The signal assignment of the major species, NOESY spectra and ^1^H spectra of **9**, for comparison, are shown in the supporting information.

2 A reviewer has pointed out that the olefin moiety (but not the dithiane) might be protonated to generate a persistent tertiary carbocation that undergoes the reaction. This hypothesis cannot be ruled out but requires an additional assumption. Thionium ion formation is indicated for the reaction **11** → **12** by the yellow color of the reaction mixture and by the fact that compounds **9** and **11** are closely related.

## References

[1] Olah GA, Prakash GKS, Sommer J (1985). Superacids.

[2] Olah GA, Brien DH, Pittman CU (1967). J Am Chem Soc.

[3] Lambert JB, Vulgaris E, Feathermann SI, Majchrzak M (1978). J Am Chem Soc.

[4] Lambert JB, Majchrzak M, Stec D (1978). J Org Chem.

[5] Guinot F, Lamaty G, Munsch H (1971) Bull Soc Chim Fr 541–546

[6] Guinot F, Lamaty G (1972). Tetrahedron Lett.

[7] Modena G, Scorrano G, Venturello P (1979). J Chem Soc Perkin Trans.

[8] Satchell DP, Satchell RS (1990). Chem Soc Rev.

[9] Föhlisch B, Haug E (1971). Chem Ber.

[10] Fabian J, Hartmann H (1973). Tetrahedron.

[11] Carlsen L, Holm A (1976). Acta Chem Scand B.

[12] Ali M, Satchell DPN, Le VT (1993). J Chem Soc Perkin Trans.

[13] Brenninger C, Pöthig A, Bach T (2017). Angew Chem In Ed.

[14] Freeman F, Angeletakis CN (1983). Org Magn Reson.

[15] Davis DG, Bax A (1985). J Magn Reson.

[16] Kütt A, Rodima T, Saame J, Raamat E, Mäemets V, Kaljurand I, Koppel IA, Garlyauskayte RY, Yagupolskii YL, Yagupolskii LM, Bernhardt E, Willner H, Leito I (2011). J Org Chem.

[17] Zhang M, Sonoda T, Mishima M, Honda T, Leito I, Koppel IA, Bonrath W, Netscher T (2014). J Phys Org Chem.

[18] Hosomi A, Sakurai H (1977). J Am Chem Soc.

[19] Mayr H, Henninger J, Siegmund T (1996). Res Chem Intermed.

