# Supplementary material for: α-Thio Carbocations (Thionium Ions) as Intermediates in Brønsted Acid-Catalyzed Reactions of Enone-Derived 1,3-Dithianes and 1,3-Dithiolanes
Source: Top Catal. 2018 Mar 5;61(7):623–9. doi: 10.1007/s11244-018-0905-6 (PMC6438600; doi:10.1007/s11244-018-0905-6)
Supplement: Supplementary file 1 — Supplementary material 1 (PDF 1740 KB) [file 11244_2018_905_MOESM1_ESM.pdf]

Table of Contents

1. General Information 1
2. Analytical Methods 2
3. Synthetic Protocols 3
4. Low Temperature NMR Experiments 13
5. Additional UV/Vis Spectra 14
6. NMR Spectra of Novel Compounds 15
7. Additional information to the NMR studies 21
8. Emission Spectra of Light Source 24
9. References 25

1. General Information

All moisture and air sensitive reactions were carried out in flame-dried glassware under an argon atmosphere using standard Schlenk techniques.

Commercially available chemicals were used without further purification, if not further mentioned. For moisture sensitive reactions tetrahydrofuran (THF) and dichloromethane (CH_2Cl_2) were purified using a MBSPS 800 *MBraun* solvent purification system. The following columns were used:

THF: 2 × MB-KOL-M type 2 (3 Å molecular sieve)

CH_2Cl_2: 2 × MB-KOL-A type 2 (aluminium oxide)

Dichloromethane for photochemical reactions and Brønsted acid-catalyzed cyclizations was additionally dried over activated molecular sieves (4 Å).

For NMR studies, *Deutero* dichloromethane-d_2 (99.6 atom% D) was employed which was dried by filtration through a pad of activated basic aluminium oxide under argon atmosphere and was stored over molecular sieves (4 Å).

The following dry solvents are commercially available and were used without further purification:

Methanol: *Acros Organics*, 99.8%, extra dry, over molecular sieve.

Technical solvents for column chromatography [pentane (P), dichloromethane (CH_2Cl_2), diethyl ether (Et_2O)] were used after simple distillation.

Flash column chromatography was performed on silica 60 (*Merck*, 230-400 mesh) with the indicated eluent mixture.

Photochemical experiments at λ = 366 nm were carried out in Duran tubes (Ø = 1.0 cm) in an RPR-100 photochemical reactor (Southern New England Ultra Violet Company, Branford, CT, USA) equipped with 16 fluorescence lamps (Philips Lighting, Black Light Blue, 8 W, λ = 366 nm). For low temperature irradiation, the reaction vessel was placed in the photo reactor for 20 minutes prior to irradiation to insure appropriate cooling of the solution.

2. Analytical Methods

Thin layer chromatography (TLC) was performed on silica coated glass plates (*Merck*, silica 60 F254) with detection by UV-light (λ = 254 nm) and/or by staining with a potassium permanganate solution [$KMnO_4$] followed by heat treatment.

$KMnO_4$-staining solution: 3.00 g potassium permanganate, 20.0 g potassium carbonate and 5.00 mL 5% sodium hydroxide solution in 300 mL water.

Infrared spectra (IR) were recorded on a *JASCO* IR-4100 spectrometer or a *Perkin Elmer* Frontier IR-FTR spectrometer by ATR technique. The signal intensities are assigned using the following abbreviations: s (strong), m (medium), w (weak), br (broad).

Nuclear magnetic resonance-spectra were recorded at room temperature either on a *Bruker* AVHD-300, AVHD-400, AVHD-500 or an AV-500 cryo. For low temperature measurements (−70 °C) a *Bruker* DRX400 was used. ^{1}H-NMR spectra were referenced to the residual solvent signal of chloroform-d_1 ($CHCl_3$ δ = 7.26 ppm), benzene-d_6 (C_6HD_5 δ = 7.16 ppm) or dichloromethane-d_2 ($CHDCl_2$ δ = 5.32 ppm). ^{13}C-NMR spectra were referenced to the ^{13}C-D triplet of $CDCl_3$ (δ = 77.2 ppm) and C_6D_6 (δ = 128.1 ppm) or to the ^{13}C-D_2 quintet of CD_2Cl_2 (δ = 54.0 ppm). Apparent multiplets which occur as a result of accidental equality of coupling constants to those of magnetically non-equivalent protons are marked as virtual (*virt.*). Following abbreviations for single multiplicities were used: *br*-broad, s-singlet, d-doublet, t-triplet, q-quartet, quin.-quintet. Assignment and multiplicity of the ^{13}C-NMR signals were determined by two dimensional NMR experiments (COSY, HSQC, HMBC, NOESY).

Mass spectroscopy (MS) was carried out on a *Agilent* MS5977A MSD spectrometer coupled to a *Agilent* 7890 B gas chromatograph using a HP-5MS UI column (30 m, 0.25 mm, 0.25 µm, 5% diphenyl- 95% dimethylpolysiloxane).

High resolution mass spectroscopy (HR-MS) was carried out on a *Thermo Scientific* DFS-HRMS spectrometer.

UV/Vis Spectroscopy was performed on a *Perkin Elmer* Lambda 35 UV/Vis spectrometer. Spectra were recorded using a *Hellma* precision cell made of quartz SUPRASIL® with a pathway of 1 mm in dry CH_2Cl_2. Concentrations are given for each spectrum.

3. Synthetic Protocols

7-(Pent-4-en-1-yl)-1,4-dithiaspiro[4.5]dec-6-ene (**4**) was synthesized according to a known procedure.[1]

Octahydro-6H-spiro[cyclopenta[1,4]cyclobuta[1,2]benzene-5,2'-[1,3]dithiolane] (5)

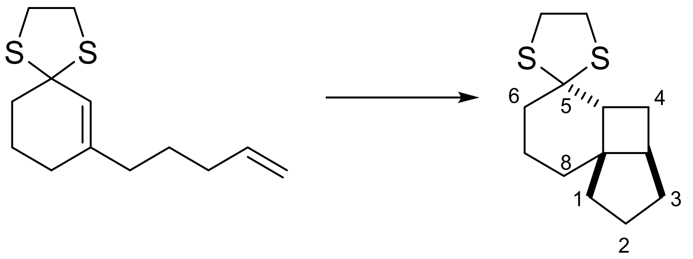

$C_{13}H_{20}S_2$
MW = 240.42 g/mol

7-(Pent-4-en-1-yl)-1,4-dithiaspiro[4.5]dec-6-ene[1] (23.4 mg, 97.3 µmol, 1.00 equiv) was dissolved in 9.7 mL CH_2Cl_2 (c = 10 mM) in a Duran tube and the solution was cooled to −40 °C. After addition of triflimide (5.71 mg, 19.6 µmol, 0.20 equiv), the solution was irradiated at λ = 366 nm (128 W) for 13.5 h. After addition of NEt_3 (13.5 µL, 97.3 mmol, 1.00 equiv) the solution was warmed to room temperature and the solvent was removed *in vacuo*. After column chromatography (SiO_2, P/Et_2O = 99/1), the title compound was obtained as a colourless oil (18 mg, 75 µmol, 77%).

TLC: R_f = 0.32 (P/Et_2O = 99/1) [$KMnO_4$].

IR (ATR): $\tilde{\nu}$ (cm^{-1}) = 2925 (s, sp^3-CH), 2848 (m, sp^3-CH), 1448 (w, sp^3-CH), 635 (w, CSC).

MS (EI, 70 eV): *m/z* (%) = 240 (88) $[M]^+$, 212 (99) $[M\text{-}C_2H_4]^+$, 179 (100) $[C_{11}H_{15}S]^+$, 171 (31), 131 (74) $[C_5H_7S_2]$, 91 (41), 79 (34).

^{1}H-NMR (500 MHz, $CDCl_3$, 298 K): δ (ppm) = 1.19 (*virt.* td, $^2J \cong {}^3J$ = 12.3 Hz, ^3J = 6.9 Hz, 1H, H-1), 1.37-1.43 (m, 1H, H-8), 1.48-1.53 (m, 1H, H-3), 1.55-1.62 (m, 2H, H-3, H-4), 1.65 (*virt.* dt, $^2J \approx\cong {}^3J$ = 13.0 Hz, ^3J = 6.6 Hz, 1H, H-1), 1.72-1.81 (m, 2H, H-2, H-7), 1.82-1.92 (m, 3H, H-2, H-7, H-8), 1.97 (ddd, ^2J = 12.9 Hz, ^3J = 9.5 Hz, ^3J = 7.5 Hz, 1H, H-4), 2.08-2.14 (m, 1H, H-6), 2.21 (*virt.* tt, $^2J \cong {}^3J \cong$ 9.0 Hz, $^3J \cong$ 4.9 Hz, 1H, H-3a), 2.33-2.41 (m, 2H, H-4a, H-6), 3.16-3.36 (m, 4H, SCH_2CH_2S).

^{13}C-NMR (126 MHz, $CDCl_3$, 300 K): δ (ppm) = 20.5 (t, C-7), 25.6 (t, C-2), 27.8 (t, C-4), 29.3 (t, C-8), 32.6 (t, C-3), 34.6 (t, C-6), 38.0 (d, C-3a), 38.1 (t, SCH_2CH_2S), 39.8 (t, SCH_2CH_2S), 40.8 (t, C-1), 48.7 (d, C-4a), 49.3 (s, C8a), 70.3 (s, C-5).

HRMS (EI, 70 eV): calculated: ($C_{13}H_{20}S_2$): 240.1001; found: 240.1000.

8-Methyl-1,5-dithiaspiro[5.5]undec-7-ene (9)

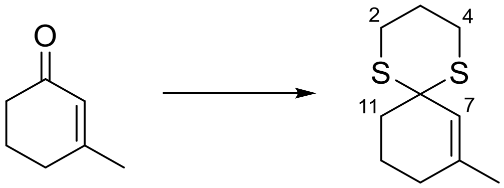

$C_{10}H_{16}S_2$
MW = 200.36 g/mol

A solution of 3-methylcyclohex-2-en-1-one (547 µL, 500 mg, 4.54 mmol, 1.00 equiv), 1,3-propandithiol (683 µL, 737 mg, 6.81 mmol, 1.50 equiv) and boron trifluoride diethyl etherate (672 µL, 773 mg, 5.45 mmol, 1.20 equiv) was stirred in dry methanol (15 mL) for two hours after which the reaction was quenched with saturated aqueous $NaHCO_3$ solution (20 mL). The biphasic mixture was extracted with Et_2O (3x15 mL) and the combined organic layers were dried over Na_2SO_4, filtered and the solvent was removed *in vacuo*. After column chromatography (SiO_2, P/Et_2O = 100/0 → 99.5/0.5) the title compound was obtained as a colourless solid in 83% yield (752 mg, 3.74 mmol, 83%).

TLC: R_f = 0.34 (P/Et_2O = 99/1) [$KMnO_4$].

M.P.: 51 °C.

IR (ATR): $\tilde{\nu}$ (cm^{-1}) = 2928 (s, sp^3-CH), 1447 (w, sp^3-CH), 1659 (w, C=C), 1273 (w), 883 (w, sp^2-CH).

MS (EI, 70 eV): *m/z* (%) = 200 (70) $[M]^+$, 126 (100) $[C_7H_{10}S]^+$, 111 (23) $[C_6H_7S]^+$, 98 (14) $[C_7H_{10}S\text{-}C_2H_4]^+$, 93 (41).

^{1}H-NMR (500 MHz, $CDCl_3$, 298 K): δ (ppm) = 1.71 (d, ^4J = 1.2 Hz, 3H, CH_3), 1.77-1.83 (m, 2H, H-10), 1.90 (dtt, ^2J = 13.8 Hz, ^3J = 10.6 Hz, ^3J = 3.2 Hz, 1H, H-3), 1.96-2.00 (m, 2H, H-9), 2.04 (dtt, ^2J = 13.8 Hz, ^3J = 6.3 Hz, ^3J = 2.9 Hz, 1H, H-3), 2.17-2.21 (m, 2H, H-11), 2.77 (ddd, ^2J = 14.6 Hz, ^3J = 6.3 Hz, ^3J = 3.2 Hz, 2H, H-2,H-4), 2.97 (ddd, ^2J = 14.6 Hz, ^3J = 10.6 Hz, ^3J = 2.9 Hz, 2H, H-2, H-4), 5.53 (d, ^4J = 1.2 Hz, 1H, H-7).

^{13}C-NMR (126 MHz, $CDCl_3$, 300 K): δ (ppm) = 19.8 (t, C-10), 24.0 (q, CH$_3$), 25.1 (t, C-3), 26.9 (t, C-2,C-4), 30.4 (t, C-9), 35.8 (t, C-11), 48.9 (s, C-6), 123.7 (d, C-7), 138.9 (s,C-8).

HRMS (EI, 70 eV): calculated: ($C_{10}H_{16}\,^{32}S_2$): 200.0688; found: 200.0686

calculated: ($C_9\,^{13}CH_{16}\,^{32}S_2$): 201.0718; found: 201.0722.

8-(4-Methylpent-4-en-1-yl)-1,5-dithiaspiro[5.5]undec-7-ene (11)

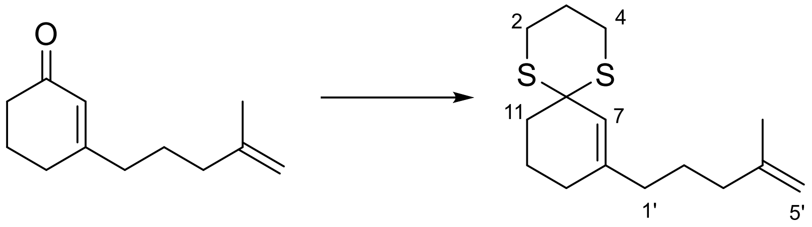

$C_{15}H_{24}S_2$
MW = 268.48 g/mol

To a solution of 3-(4-Methylpent-4-en-1-yl)cyclohex-2-en-1-one (150 mg, 841 µmol, 1.00 equiv) in dry MeOH (4.6 mL) were added 1,3-propandithiol (127 µL, 137 mg, 1.26 mmol, 1.20 equiv) and $BF_3{\cdot}OEt_2$ (125 µL, 143 mg, 1.01 mmol, 1.20 equiv). The solution was stirred for four hours at room temperature and following this, the reaction was quenched with saturated aqueous $NaHCO_3$ solution (10 mL). The biphasic mixture was extracted with Et_2O (3x15 mL) and the combined organic layers were dried over Na_2SO_4, filtered and the solvent was removed *in vacuo*. After column chromatography (SiO_2, P/Et_2O = 99/1) the title compound was obtained as a colourless oil (130 mg, 488 µmol, 58%).

TLC: R_f = 0.29 (P/Et_2O = 99/1) [$KMnO_4$].

IR (ATR): $\tilde{\nu}$ (cm^{-1}) = 3071 (w, sp^2-CH), 2932 (s, sp^3-CH), 1649 (m, C=C), 1423 (m, sp^3-CH), 1273 (m), 884 (s, C-S).

MS (EI, 70 eV): *m/z* (%) = 268 (46) $[M]^+$, 213 (5) $[M-C_4H_7]^+$, 193 (28) $[C_{12}H_{18}S]^+$, 179 (100) $[C_{12}H_{18}S-CH_3]^+$, 139 (54) $[C_{12}H_{18}S-C_4H_7]^+$.

^{1}H-NMR (500 MHz, C_6D_6, 298 K): δ (ppm) = 1.42 (*virt.* quint, $^3J \cong 7.6$ Hz, 2H, H-2’), 1.46-1.54 (m, 1H, H-3), 1.58 (s, 3H, CH_3), 1.63 (ddd, ^2J = 14.2 Hz, ^3J = 6.4 Hz, ^3J = 3.1 Hz, 1H, H-3), 1.74-1.82 (m, 6H, H-9, H-10, H-1’), 1.87 (t, ^3J = 7.5 Hz, 2H, H-3’), 2.19-2.25 (m, 2H, H-11), 2.31 (ddd, ^2J = 14.5 Hz, ^3J = 6.4 Hz, ^3J = 3.2 Hz, 2H, H-2, H-4), 2.60 (ddd, ^2J = 14.5 Hz, ^3J = 11.2 Hz, ^3J = 3.1 Hz, 2H, H-2, H-4), 4.77 (*br* s, 1H, H-5’), 4.78 (*br* s, 1H, H-5’), 5.79 (*br* s, 1H, H-7).

^{13}C-NMR (126 MHz, C_6D_6, 300 K): δ (ppm) = 20.1 (t, C-10), 22.2 (q, CH_3), 25.0 (t, C-3), 25.3 (t, C-2‘), 26.7 (t, C-2, C-4), 28.7 (t, C-9), 36.2 (t, C-11), 37.2 (t, C-1‘), 37.3 (t, C-3‘), 49.3 (s, C-6), 110.5 (t, C-5‘), 124.4 (d, C-7), 142.0 (s, C-8), 145.3 (s, C-4‘).

HRMS (EI, 70 eV): calculated: ($C_{15}H_{24}{}^{32}S_2$): 268.1314; found: 268.1308;

calculated: ($C_{14}{}^{13}CH_{24}{}^{32}S_2$): 269.1348; found: 269.1343.

10-Methyl-1,5-dithiadispiro[5.1.5⁸.3⁶]hexadec-10-ene (12)

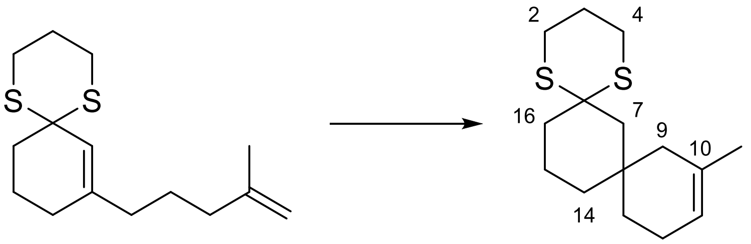

$C_{15}H_{24}S_2$
MW = 268.48 g/mol

8-(4-Methylpent-4-en-1-yl)-1,5-dithiaspiro[5.5]undec-7-ene (23.0 mg, 85.7 µmol, 1.00 equiv) was dissolved in dry CH_2Cl_2 (8.6 mL) and the resulting mixture cooled to −78 °C. Following the addition of 1,1,2,2,3,3-hexafluoropropane-1,3-disulfonimide (1.88 mg, 6.43 µmol, 0.10 equiv) the solution was stirred for 5.5 h at −78 °C in the dark, after which the reaction was quenched *via* addition of triethyl amine (11.9µL, 84.7 µmol, 1.00 equiv). The solution was allowed to warm to room temperature and the solvent was removed *in vacuo*. After column chromatography (SiO_2, P/Et_2O = 99.5/0.5 → 99/1) the title compound was obtained as a colourless oil (20.1 mg, 73.7 µmol, 87%).

TLC: R_f = 0.35 (P/Et_2O = 98/2) [$KMnO_4$].

IR (ATR): $\tilde{\nu}$ (cm^{-1}) = 2926 (s, sp^3-CH), 1444 (m, sp^3-CH), 1275 (w), 795 (w, sp^3-CH).

MS (EI, 70 eV): *m/z* (%) = 268 (100) $[M]^+$, 200 (52) $[M-C_5H_8]^+$, 159 (60), 118 (62), 105 (58), 91 (32).

^{1}H-NMR (500 MHz, $CDCl_3$, 298 K): δ (ppm) = 1.28 (ddd, ^2J = 13.1 Hz, ^3J = 9.3 Hz, ^3J = 3.7 Hz, 1H, H-14), 1.36-1.46 (m, 2H, H-13, H14), 1.53-1.58 (m, 1H, H-13), 1.62 (*br* s, 3H, CH_3), 1.60-1.73 (m, 2H, H-15, H-15), 1.78 (d, ^2J = 14.4 Hz, 1H, H-9), 1.82-1.95 (m, 3H, H-3, H-9, H-12), 1.96-2.09 (m, 5H, H-3, H-7, H-7, H-12, H-16), 2.17 (ddd, ^2J = 13.0 Hz, ^3J = 6.9 Hz, ^3J = 3.3 Hz, 1H, H-16), 2.68-2.79 (m, 2H, H-2, H-4), 2.89-2.99 (m, 2H, H-2, H-4), 5.32 (*virt.* tq, $^3J \cong$ 3.2 Hz, ^4J = 1.6 Hz, 1H, H-11).

^{13}C-NMR (126 MHz, $CDCl_3$, 300 K): δ (ppm) = 19.1 (t, C-15), 22.7 (t, C-7), 24.2 (q, CH_3), 25.8 (t, C-3), 26.7 (t, C-2), 26.8 (t, C-4), 33.6 (s, C-8), 35.1 (t, C-13), 36.9 (t, C-14), 38.6 (t, C-16), 42.9 (t, C-12), 47.3 (t, C-9), 49.9 (s, C-6), 119.7 (d, C-11), 132.5 (s, C-10).

HRMS (EI, 70 eV): calculated: ($C_{15}H_{24}{}^{32}S_2$): 268.1314; found: 268.1309;

calculated: ($C_{14}{}^{13}CH_{24}{}^{32}S_2$): 269.1348; found: 269.1343.

4-Methylpent-4-enoic acid (16)

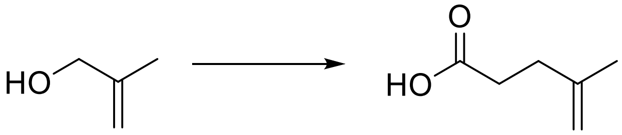

$C_6H_{10}O_2$
MW = 114.14 g/mol

A mixture of 2-methylprop-2-en-1-ol (4.67 mL, 4.00 g, 55.5 mmol, 1.00 equiv), trimethyl orthoacetate (11.0. mL, 10.0 g, 83.2 mmol, 1.50 eq.) and propionic acid (415 µl, 411 mg, 5.55 mmol, 0.10 eq.) was stirred at 100 °C until complete removal of MeOH *via* distillation was observed. Subsequently, the solution was stirred for additional 15 hours at 145 °C. After cooling to room temperature, the organic layer was washed with saturated aqueous $NaHCO_3$ solution (3x30 mL) and was then dried over Na_2SO_4. After filtration and removal of volatile compounds *in vacuo* the resulting intermediate was dissolved in THF/H_2O (v/v = 1/1), LiOH (2.66 g, 111 mmol, 2.00 equiv) was added and the reaction mixture was stirred at room temperature for 3 hours. Following this, the reaction mixture was washed with Et_2O (5x20 mL). The aqueous layer was acidified to a pH of 1 *via* dropwise addition of conc. aqueous HCl solution at 0 °C. The title compound **16** was obtained after extraction with CH_2Cl_2 (5x20 mL), drying over Na_2SO_4 and removal of volatiles *in vacuo* as a colourless oil in (2.6 g, 23mmol, 41%).

TLC: R_f = 0.52 (P/EtOAc = 3/1) [$KMnO_4$].

^{1}H-NMR (500 MHz, $CDCl_3$, 298 K): δ (ppm) = 1.75 (s, 3H, CH_3), 2.34 (t, ^3J = 7.8 Hz, 2H, H-3), 2.52 (t, ^3J = 7.8 Hz, 2H, H-2), 4.71 (*br* s, 1H, H-5), 4.77 (*br* s, 1H, H-5).

^{13}C-NMR (126 MHz, $CDCl_3$, 300 K): δ (ppm) = 22.7 (q, CH_3), 32.3 (t, C-3), 32.5 (t, C-2), 110.7 (t, C-5), 143.9 (s, C-4), 179.9 (s, C-1).

The obtained data matched that previously reported in the literature[2]

4-Methylpent-4-en-1-ol (17)

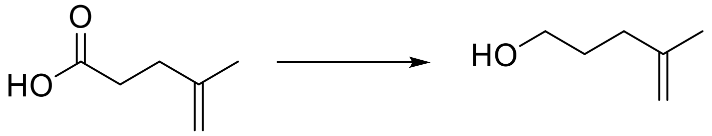

$C_6H_{12}O$
MW = 100.09 g/mol

To a precooled (0°C) suspension of lithium aluminium hydride (943 mg, 24.8 mmol, 1.02 equiv) in dry THF (40 mL) was slowly added a solution of 4-methylpent-4-enoic acid (**16**) (2.60 g, 24.6 mmol, 1.00 equiv) in dry THF (10 mL). The mixture was allowed to warm to room temperature and was subsequently stirred for 21 hours, before it was recooled to 0 °C. Following this water (1.5 mL), 10% aqueous NaOH solution (3.0 mL) and water (1.5 mL) were added to the reaction mixture. The precipitate removed *via* filtration and the aqueous layer was extracted with Et_2O (4x30 mL). After drying over Na_2SO_4, filtration and removal of the solvent *in vacuo* the title compound **17** was obtained as a colourless oil in 78% yield (1.83 g, 19.2 mmol).

TLC: R_f = 0.33 (P/Et_2O = 3/1) [$KMnO_4$].

^{1}H-NMR (400 MHz, $CDCl_3$, 298 K): δ (ppm) = 1.67-1.76 (m, 2H, H-2), 1.74 (t, ^4J = 1.1 Hz, 3H, C*H*3), 2.10 (td, ^3J = 7.6 Hz, ^4J = 1.1 Hz, 2H, H-3), 3.66 (t, ^3J = 6.5 Hz, 2H, H-1), 4.70-4.74 (m, 2H, H-5).

^{13}C-NMR (101 MHz, $CDCl_3$, 300 K): δ (ppm) = 22.5 (q, *C*H3), 30.7 (t, C-2), 34.3 (t, C-3), 62.9 (t, C.1), 101.4 (t, C-5), 145.7 (s, C-4).

The obtained data matched that previously reported in the literature[3]

5-Bromo-2-ethylpent-1-ene (18)

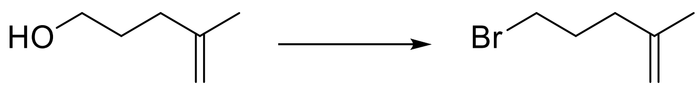

$C_6H_{11}Br$
MW = 163.06 g/mol

A solution of 4-methylpent-4-en-1-ol (**17**) (1.80 g, 18.0 mmol, 1.00 equiv) and triethyl amine (3.24 mL, 2.36 g, 23.4 mmol, 1.30 equiv) in dry THF (40 mL) was cooled to 0 °C and methanesulfonyl chloride (1.81 mL, 2.68 g, 23.4 mmol, 1.30 equiv) was then added dropwise. The solution was allowed to warm to room temperature and was stirred for four hours. After addition of water (60 mL), the aqueous layer was extracted with CH_2Cl_2 (3x45 mL). The combined organic layers were dried over Na_2SO_4, filtered and the solvent was removed *in vacuo*. The crude mesylate was used without further purification and was dissolved in acetone

(55 mL). After addition of lithium bromide (4.68 g, 53.9 mmol, 3.00 equiv), the mixture was stirred at 60 °C for 12 h. After cooling to room temperature, water (30 mL) was added and the aqueous layer was extracted with Et_2O (3x30 mL). The combined organic layers were dried over Na_2SO_4, filtered and the solvent was removed *in vacuo*. After purification by column chromatography (SiO_2, P), the title compound **18** was obtained as a colourless oil (1.65 g, 9.88 mmol, 55%).

TLC: R_f = 0.92 (P) [$KMnO_4$].

^{1}H-NMR (400 MHz, $CDCl_3$, 298 K): δ (ppm) = 1.73 (s, 3H, C*H*3), 2.00 (*virt.* q, $^3J \cong 6.7$ Hz, 2H, H-4), 2.16 (t, $^3J = 7.4$ Hz, 2H, H-3), 3.41 (t, $^3J = 6.7$ Hz, 2H, H-5), 4.71-4.73 (m, 1H, H-1_b), 4.76 (*br* s, 1H, H-1_a).

^{13}C-NMR (101 MHz, $CDCl_3$, 300 K): δ (ppm) = 22.5 (q, *C*H3), 30.7 (t, C-4), 33.5 (t, C-5), 36.2 (t, C-3), 111.2 (t, C-1), 144.1 (s, C-2).

The obtained data matched that previously reported in the literature.[4]

3-(4-Methylpent-4-en-1-yl)cyclohex-2-en-1-one (19)

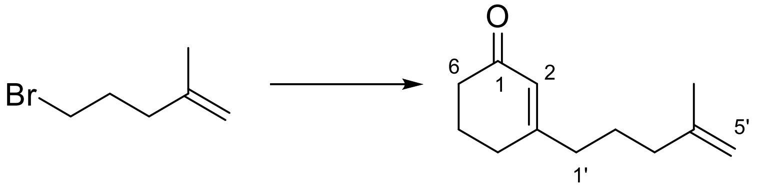

$C_{12}H_{18}O$
MW = 178.28 g/mol

To a suspension of magnesium (90.2 mg, 3.71 mmol, 1.30 equiv) and a catalytic amount of iodine in dry THF (1 mL) was added a solution of 5-bromo-2-ethylpent-1-ene (**18**) (630 mg, 3.86 mmol, 1.35 equiv) in dry THF (5.8 mL). After full consumption of magnesium was observed, a solution of 3-ethoxy-2-cyclohexenone (415 µL, 400 mg, 2.85 mmol, 1.00 equiv) in dry THF (4.5 mL) was added slowly. After stirring for four hours at room temperature, saturated aqueous NH_4Cl solution (16 mL) was added. The aqueous layer was extracted with Et_2O (3x25 mL). The combined organic layers were dried over Na_2SO_4, filtered and the solvent was removed *in vacuo*. After purification by column chromatography (SiO_2, P/Et_2O = 4/1) the title compound **19** was obtained as a colourless oil (320 mg, 1.80 mmol, 63%).

TLC: R_f = 0.31 (P/Et_2O = 4/1) [UV/$KMnO_4$].

IR (ATR): $\tilde{\nu}$ (cm^{-1}) = 2935(m, sp^2-CH), 1671 (s, C=O), 1626 (m, C=C), 1455 (w, sp^3-CH), 1374 (w, sp^3-CH), 1325 (w, sp^2-CH).

MS (EI, 70 eV): *m/z* (%) = 178 (16) $[M]^+$, 163 (21) $[M-CH_3]^+$, 150 (7), $[M-CH_2H_4]^+$, 135 (38), 123 (100) $[M-C_4H_7]^+$, 110 (86), 91 (80), 82 (98) $[M-C_2H_4-C_5H_9]^+$.

^{1}H-NMR (500 MHz, $CDCl_3$, 298 K): δ (ppm) = 1.61-.167 (m, 2H, H-2'), 1.71 (s, 3H, C*H*3), 1.97-2.05 (m, 4H, H-5, H-3'), 2.20 (t, ^3J = 7.9 Hz, 2H, H-1'), 2.28 (td, ^{3}J = 5.9 Hz, ^4J = 0.8 Hz, 2H, H-4), 2.34-2.38 (m, 2H, H-6), 4.67-4.64 (m, 1H, H-5'), 4.73-4.75 (m, 1H, H-5'), 5.89 (*virt*. q, $^4J \cong$ 1.3 Hz, 1H, H-2).

^{13}C-NMR (126 MHz, $CDCl_3$, 300 K): δ (ppm) = 22.4 (q, *C*H3), 22.9 (t, C-5), 24.8 (t, C-2'), 29.9 (t, C-4), 37.3 (t, C-3'), 37.5 (C-6), 37.6 (C-1'), 110.7 (t, C-5'), 125.9 (d, C-2), 145.1(s, C-4'), 166.5 (s, C-3), 200.1 (s, C-1).

HRMS (EI, 70 eV): calculated: ($C_{12}H_8O$): 178.1352; found: 178.1355;

calculated: ($C_{11}{}^{13}CH_{18}O$): 179.1386; found: 179.1393.

3-Pentylcyclohex-2-en-1-one (20)

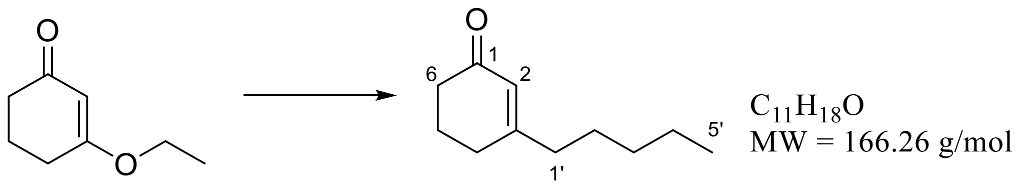

In a flame dried flask magnesium (256 mg, 9.27 mmol, 1.30 equiv) and a catalytic amount of iodine were suspended in dry THF (1 mL). Following this, a solution of 1-bromopentane (1.19 mL, 1.45 g, 9.27 mmol, 1.35 equiv) in THF (12 mL) was added dropwise. After stirring at room temperature for one hour a solution of 3-ethoxycyclohex-2-en-1-one (1.04 mL, 1.00 g, 7.13 mmol, 1.00 equiv) and THF (8 mL) was added. The reaction was quenched after 46 hours *via* addition of 25 mL saturated aqueous NH_4Cl solution. The aqueous phase was then extracted three times with Et_2O (25 mL). The combined organic layers were dried over Na_2SO_4, filtered and the solvent was removed *in vacuo*. After column chromatography (SiO_2, P/Et_2O = 2/1), the title compound **20** was obtained as a colourless oil (595 mg, 3.58 mmol, 50%).

TLC: R_f = 0.40 (P/Et_2O = 2/1) [UV/$KMnO_4$].

^{1}H NMR (300 MHz, $CDCl_3$, 300 K): δ (ppm) = 0.88 (t, ^3J = 6.8 Hz, 3H, H-5'), 1.21-1.40 (m, 4H, H-3', H-4'), 1.50 (*virt*. quin, $^3J \cong$ 7.5 Hz, 2H, H-2'), 1.99 (*virt*. quint, $^3J \cong$ 6.4 Hz, 2H, H-5),

2.20 (t, ^3J = 7.7 Hz, 2H, H-1'), 2.28 (t, ^3J = 6.8 Hz, 2H, H-4), 2.32-2.39 (m, 2H, H-6), 5.87 (*virt.* quint, $^4J \cong$ 1.3 Hz, 1H, H-1).

^{13}C NMR (76 MHz, $CDCl_3$, 300 K): δ (ppm) = 14.1 (q, C-5'), 22.6 (t, C-4'), 22.9 (t, C-5), 26.8 (t, C-2'), 29.8 (t, C-4), 31.6 (t, C-3'), 37.5 (t, C-6), 38.2 (t, C-1'), 125.8 (d, C-2), 166.9 (s, C-3), 200.1(s, C-1).

The obtained data matched that previously reported in the literature.[5]

8-Pentyl-1,5-dithiaspiro[5.5]undec-7-ene (21)

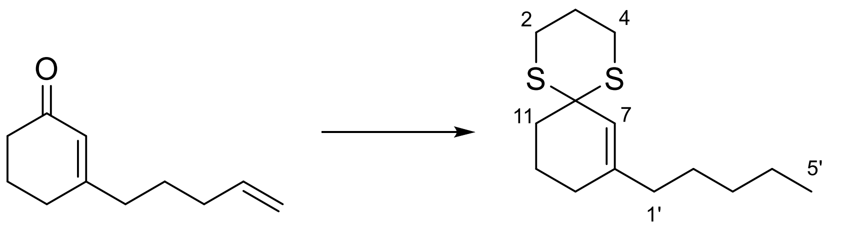

$C_{14}H_{24}S_2$
MW = 256.13 g/mol

To a solution of 3-pentylcyclohex-2-en-1-one (**20**) (405 mg, 2.44 mmol, 1.00 equiv) in dry MeOH (5.3 mL) in a flame dried flask were added 1,3-propandithiol (360 µL, 391 mg, 3.61 mmol, 1.50 equiv) and boron trifluoride diethyl etherate (365 µL, 410 mg, 2.89 mmol, 1.20 equiv). After stirring for three hours, the reaction mixture was quenched *via* addition of saturated aqueous $NaHCO_3$ solution (4.2 mL). The aqueous phase was extracted with Et_2O (3x35 mL). The combined organic layers were dried over Na_2SO_4, filtered and the solvent was removed in vacuo. Following purification by column chromatography (SiO_2, P/Et_2O = 99/1) afforded the title compound was afforded **21** as a colourless oil (529 mg, 2.06 mmol, 84%).

TLC: R_f = 0.33 (P/Et_2O = 99/1) [$KMnO_4$].

IR (ATR): $\tilde{\nu}$ (cm^{-1}) = 2926 (s, sp^3-CH), 1653 (w, C=C), 1422 (m, sp^3-CH), 1273(m), 885(s).

MS (EI, 70 eV): *m/z* (%) = 256 (100) $[M]^+$, 192 (90) $[C_{11}H_{18}S_1]^+$, 149 (40), 139 (84), 126 (65).

^{1}H NMR (500 MHz, $CDCl_3$, 298 K): δ (ppm) = 0.88 (t, ^3J = 7.1 Hz, 3H. H-5'), 1.22-1.35 (m, 4H, H-3', H-4'). 1.41 (*virt.* q, $^3J \cong$ 7.5 Hz, 2H, H-2'), 1.74-1.81 (m, 2H, H-10), 1.88 (dtt, ^2J = 14.4 Hz, ^3J = 10.9 Hz, ^3J = 3.2 Hz, 1H, H-3), 1.95-2.01 (m, 4H, H-9 H-1'), 2.05 (dtt, ^2J = 14.4 Hz, ^3J = 5.9 Hz, ^3J = 2.8 Hz, 1H, H-3), 2.19-2.24 (m, 2H, H-11), 2.74 (ddd, ^2J = 14.3 Hz, ^3J = 5.9 Hz, ^3J = 3.2 Hz, 2H, H-2,H-4), 2.98 (ddd, ^2J = 14.3 Hz, ^3J = 10.9 Hz, ^3J = 2.8 Hz, 2H, H-2,H-4), 5,48 (s, 1H, H-7).

^{13}C NMR (126 MHz, $CDCl_3$, 300 K): δ (ppm) = 14.2 (q, C-5‘), 20.0 (t, C-10), 22.7 (t, C-4‘), 25.1 (t, C-3), 27.0 (t, C-2, C-4), 27.2 (t, C-2‘), 28.8 (t, C-9), 31.6 (t, C-3‘), 36.0 (t, C-11), 37.7 (t, C-1‘), 49.0 (s, C-6), 123.0 (d, C-7) 143.0 (s,C-8).

HRMS (EI, 70 eV): calculated: ($C_{14}H_{24}{}^{32}S_2$): 256.1314; found: 256.1315;

calculated: ($C_{13}{}^{13}CH_{24}{}^{32}S_2$): 257.1348; found:257.1346.

4. Low Temperature NMR Experiments

For ^{1}H-NMR experiments Tf_2NH (43.9 mg, 156 μmol, 6.25 equiv) was added to an NMR tube under argon atmosphere using a glovebox, which was then cooled to −78 °C outside the glovebox. Under an argon atmosphere the added $Tf_{2\#}NH$ was dissolved in 0.7 mL dry CD_2Cl_2 and 8-methyl-1,5-dithiaspiro[5.5]undec-7-ene (**9**, 5.00 mg, 25.0 μmol, 1.00 equiv) was added. After sufficient mixing the yellow-orange solution was warmed to 203 K (−70 °C) in a *Bruker* DRX400 spectrometer and spectra were recorded at this temperature.

For ^{13}C and 2D experiments dithiane **9** (20.0 mg, 100 μmol, 1.00 equiv) and Tf_2NH (175 mg, 624 μmol, 6.25 equiv) were dissolved in 0.6 ml dry CD_2Cl_2.

5. Additional UV/Vis Spectra

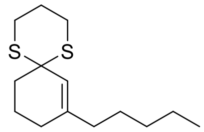

21

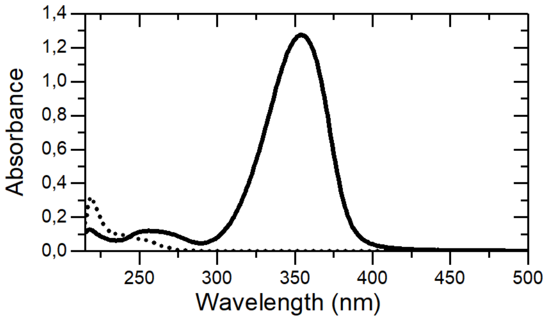

Addition of 12.5 equiv Tf_2NH: λ_{max} = 356 nm, $\varepsilon_{356\ nm}$ = 25534 $Lmol^{-1}cm^{-1}$ (c = 0.5 mM).

6. NMR Spectra of Novel Compounds

Octahydro-6H-spiro[cyclopenta[1,4]cyclobuta[1,2]benzene-5,2'-[1,3]dithiolane] (5)

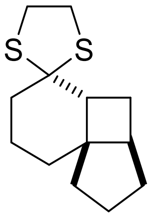

^{1}H-NMR (500 MHz, $CDCl_3$, 298 K)

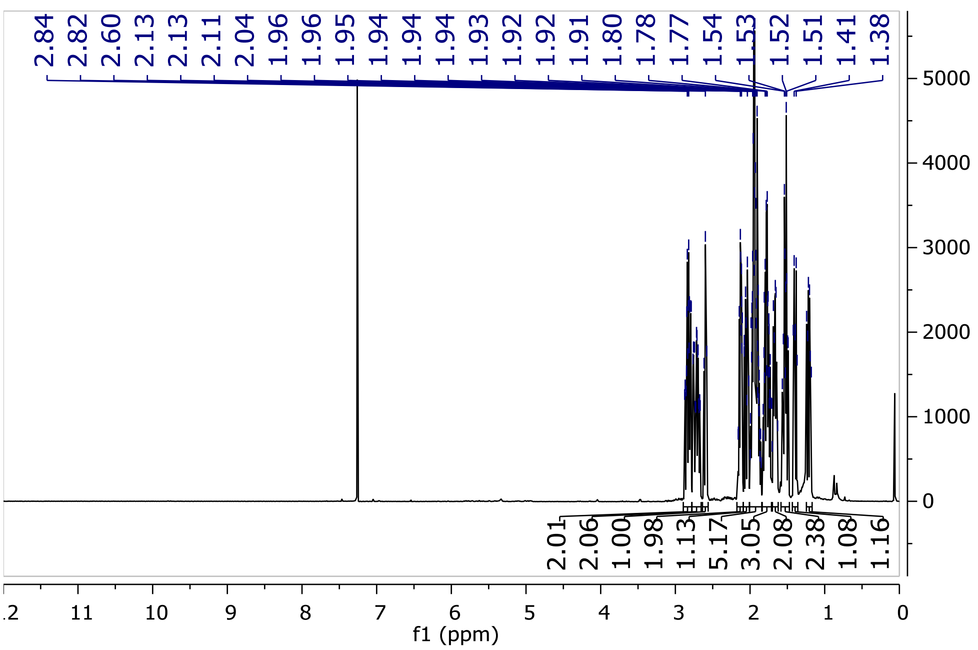

^{13}C-NMR (126 MHz, $CDCl_3$, 300 K)

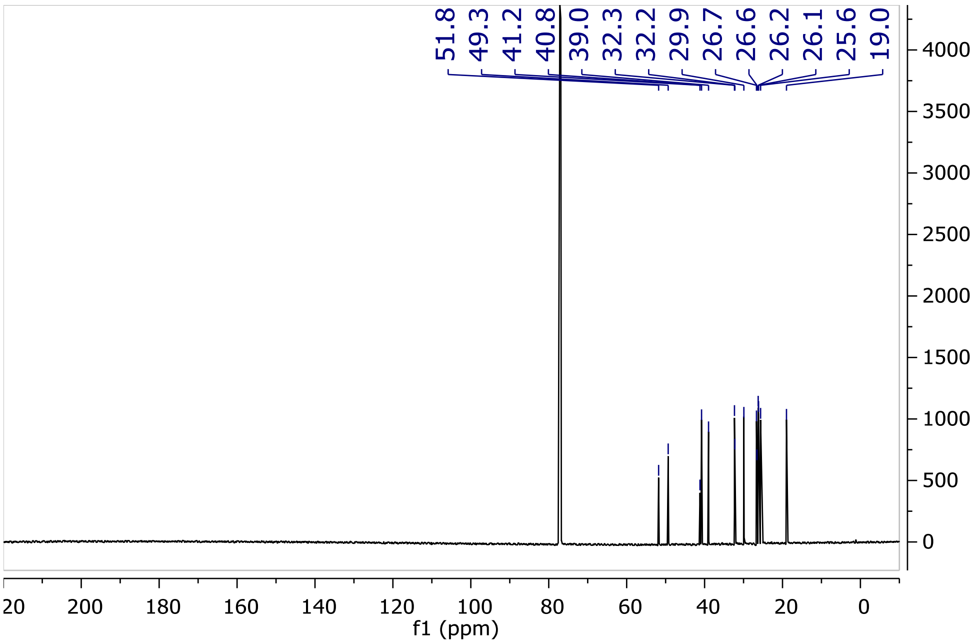

8-Methyl-1,5-dithiaspiro[5.5]undec-7-ene (9)

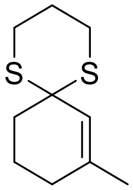

^{1}H-NMR (500 MHz, $CDCl_3$, 298 K)

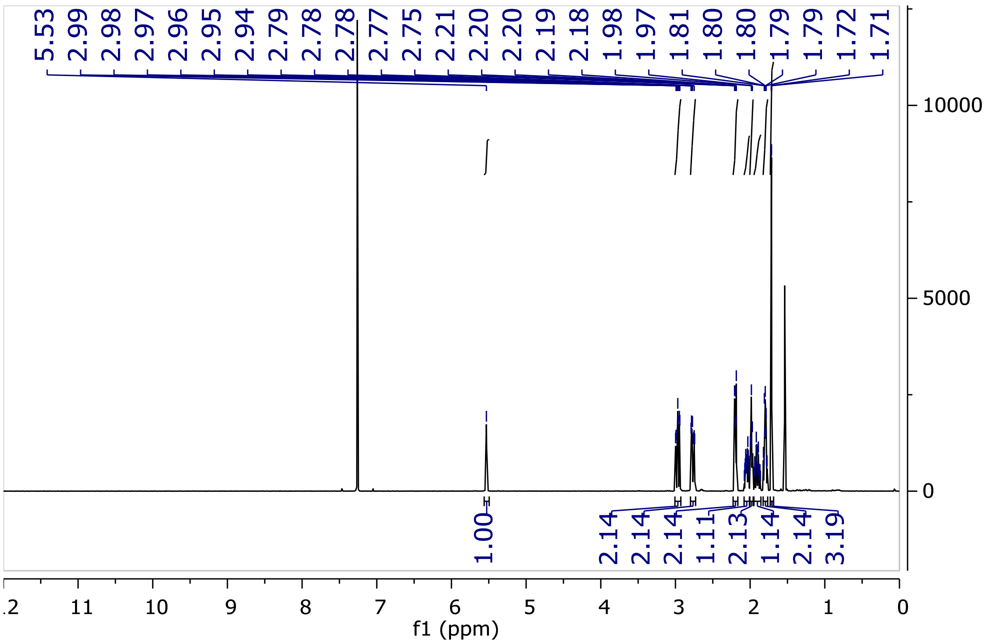

^{13}C-NMR (126 MHz, $CDCl_3$, 300 K)

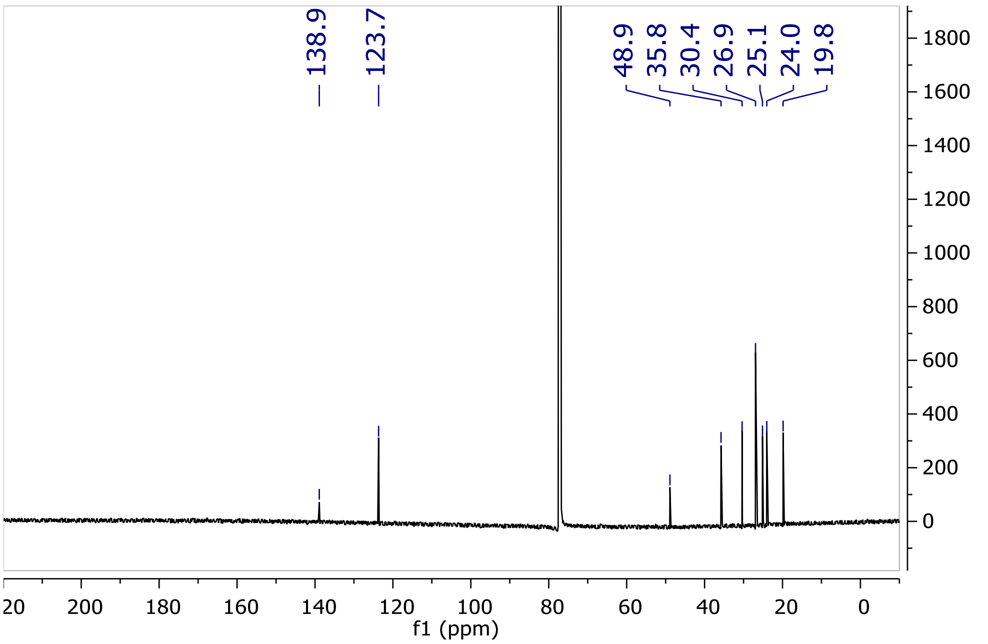

8-(4-Methylpent-4-en-1-yl)-1,5-dithiaspiro[5.5]undec-7-ene (11)

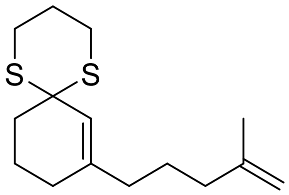

^{1}H-NMR (500 MHz, $CDCl_3$, 298 K)

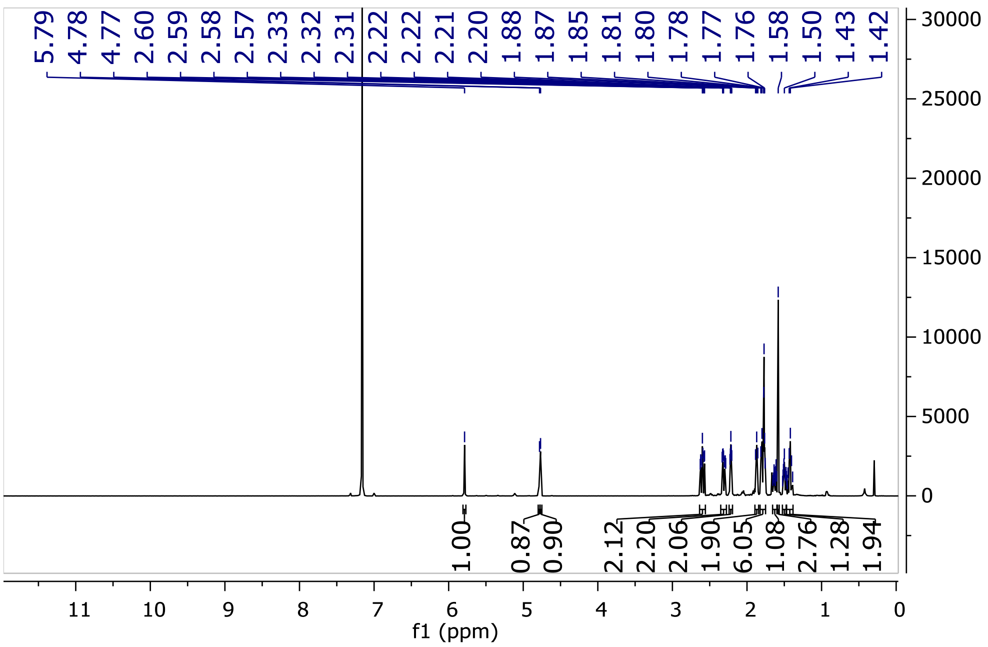

^{13}C-NMR (126 MHz, $CDCl_3$, 300 K)

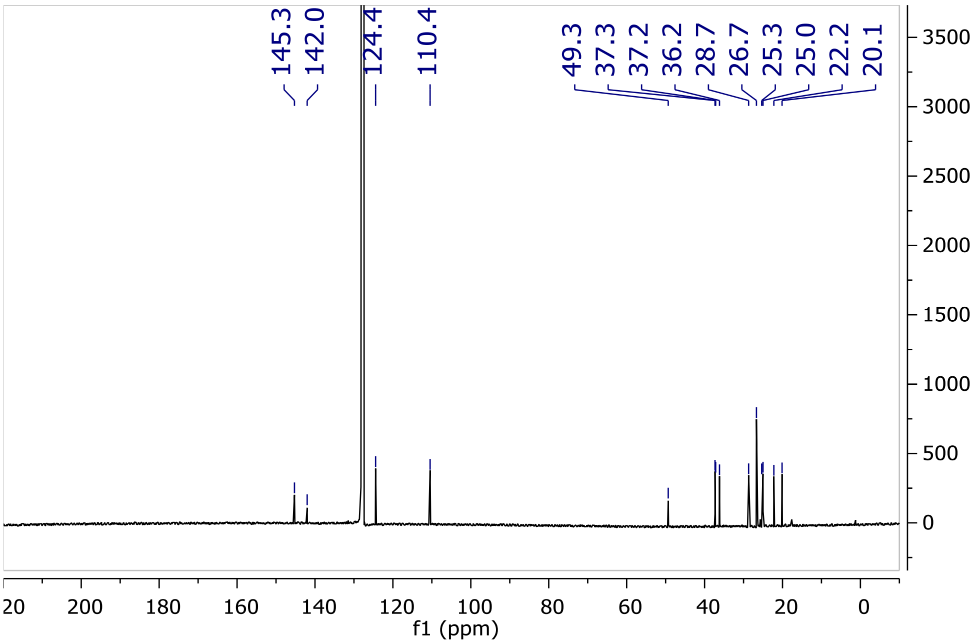

10-Methyl-1,5-dithiadispiro[5.1.58.36]hexadec-10-ene (12)

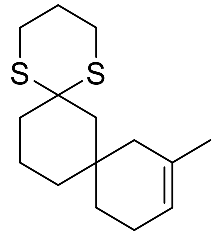

^{1}H-NMR (500 MHz, $CDCl_3$, 298 K)

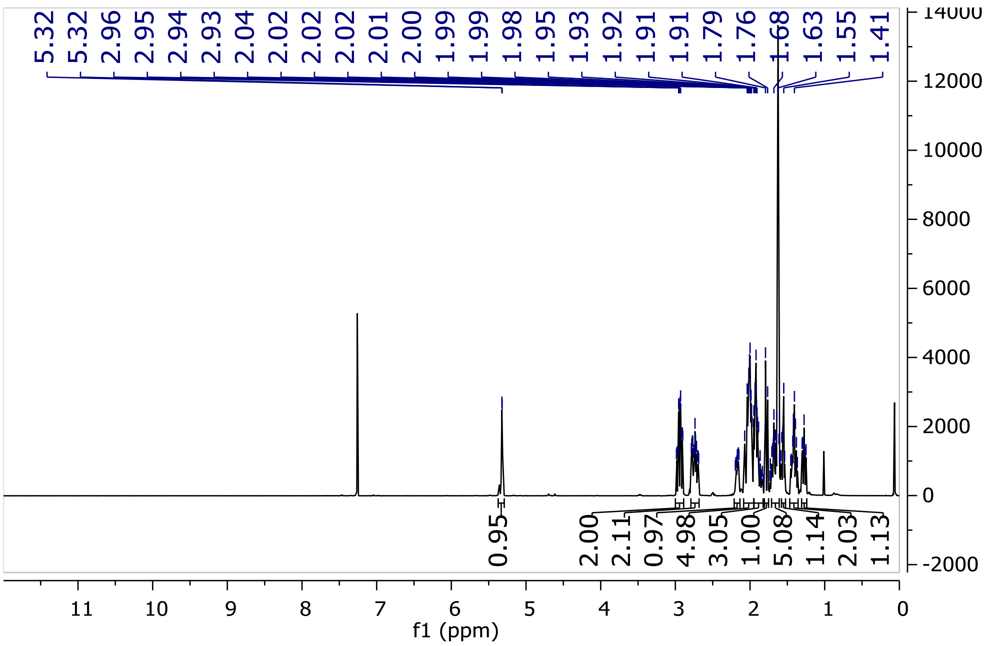

^{13}C-NMR (126 MHz, $CDCl_3$, 300 K)

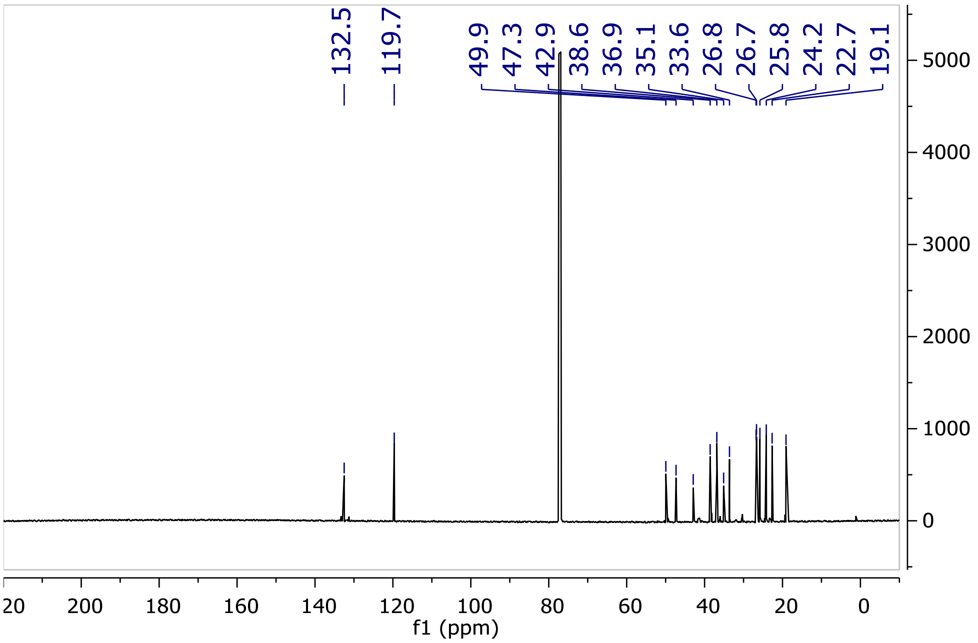

3-(4-Methylpent-4-en-1-yl)cyclohex-2-en-1-one (19)

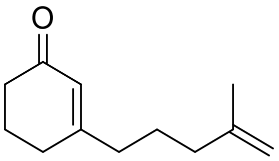

^{1}H-NMR (500 MHz, $CDCl_3$, 298 K)

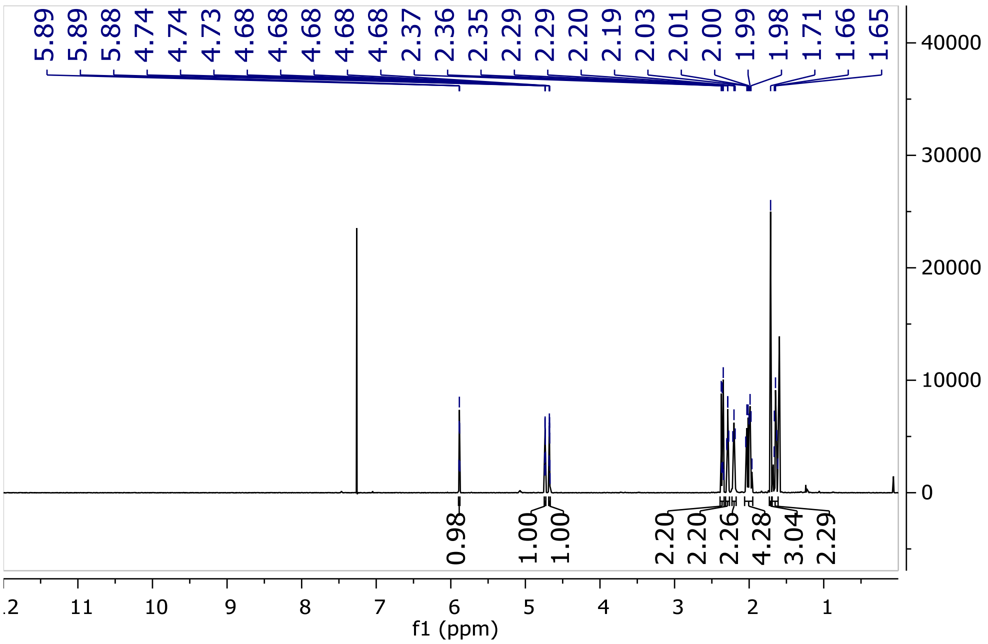

^{13}C-NMR (126 MHz, $CDCl_3$, 300 K)

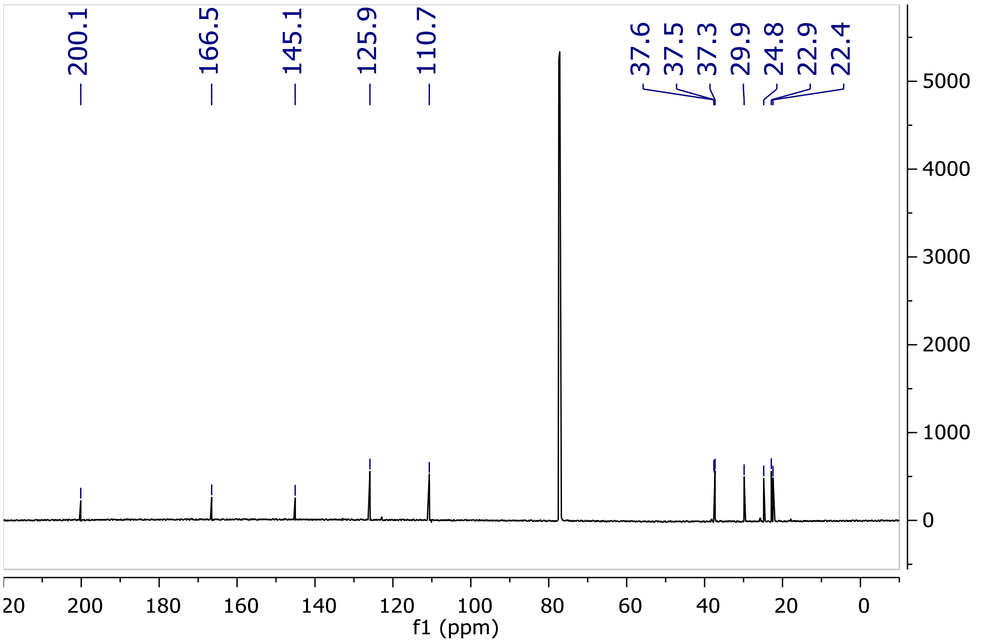

8-Pentyl-1,5-dithiaspiro[5.5]undec-7-ene (21)

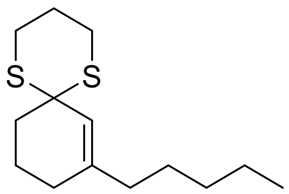

^{1}H-NMR (500 MHz, $CDCl_3$, 298 K)

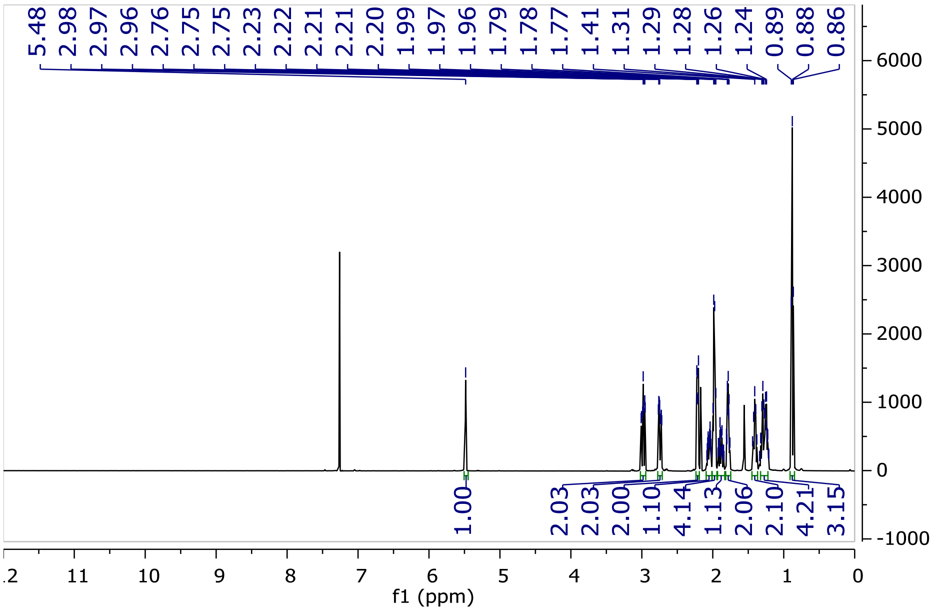

^{13}C-NMR (126 MHz, $CDCl_3$, 300 K)

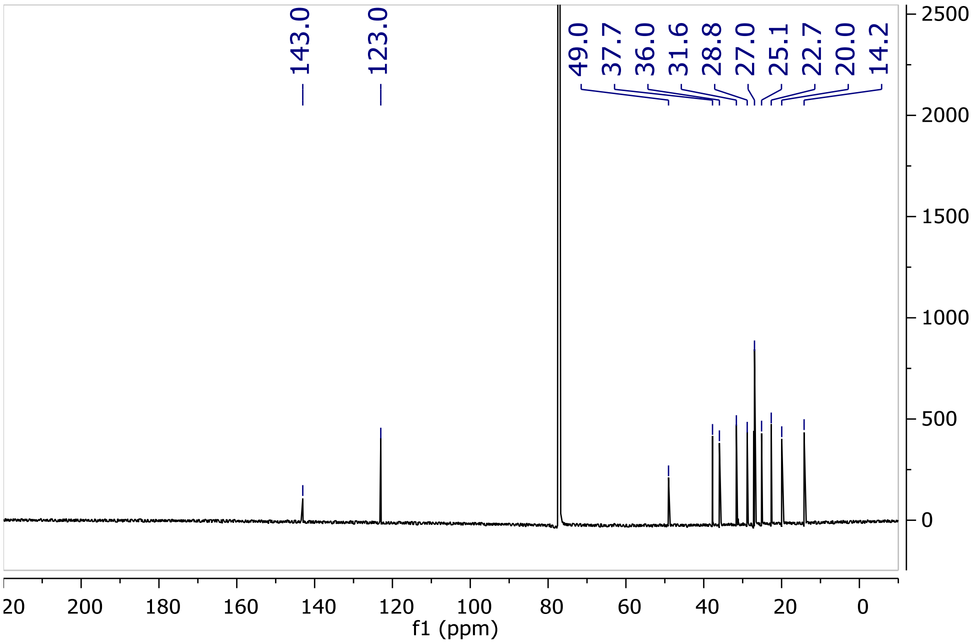

7. Additional information to the NMR studies

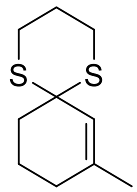

^{1}H-NMR (500 MHz, CD_2Cl_2, 298 K)

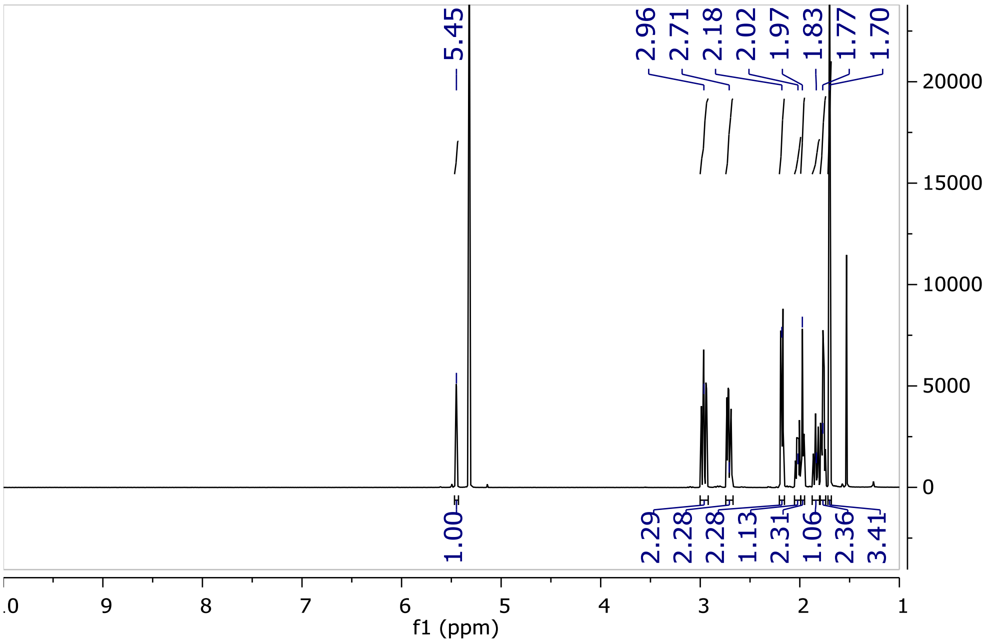

^{1}H-NMR (400 MHz, CD_2Cl_2, 203 K)

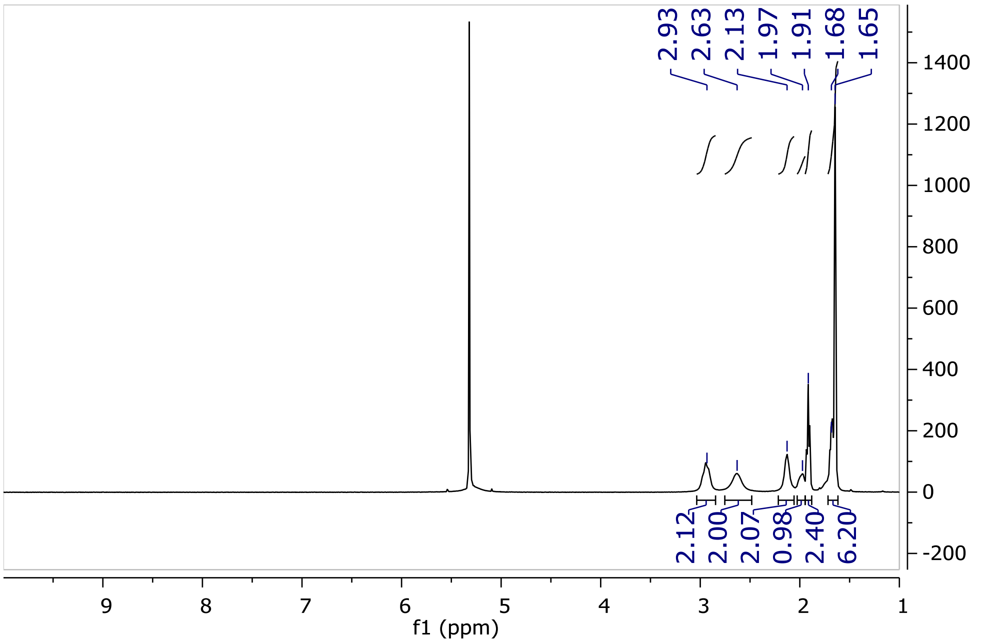

Signal assignment of **10**a:

shift of hydrogen (italic) and carbon atoms in ppm; * assignment is interconvertible

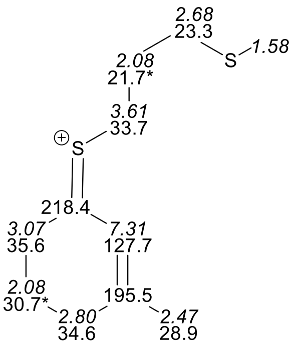

10a

Important NOE contacts

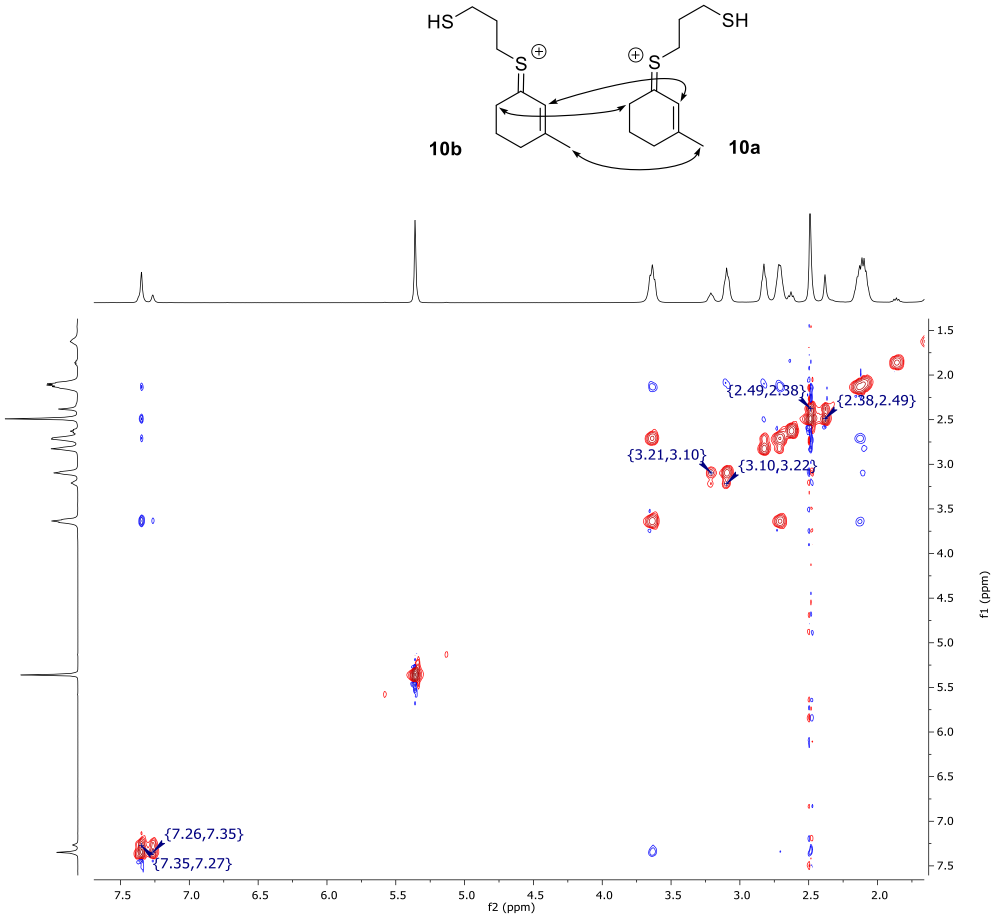

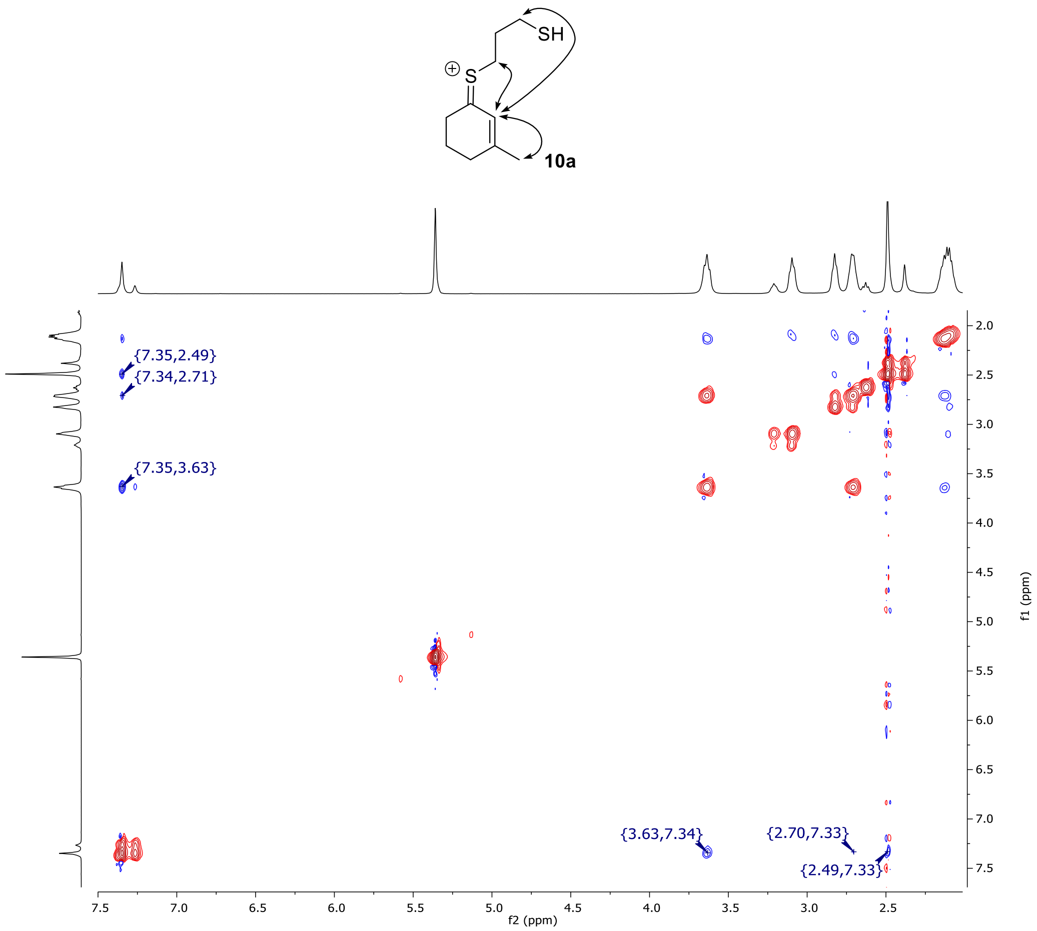
SH
S
10a
{7.35,2.49}
{7.34,2.71}
{7.35,3.63}
{3.63,7.34}
{2.70,7.33}
{2.49,7.33}
f1 (ppm)
f2 (ppm)

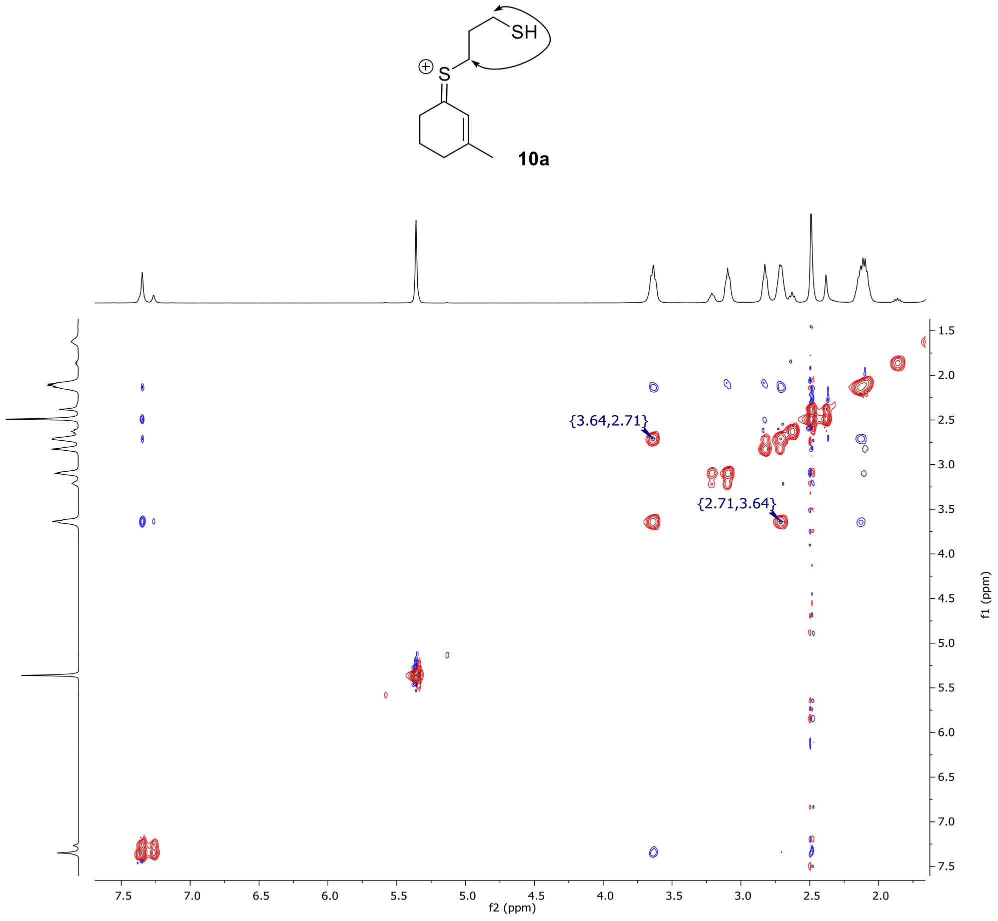
SH
S
10a
{3.64,2.71}
{2.71,3.64}
f1 (ppm)
f2 (ppm)

8. Emission Spectra of Light Source

Philips Lighting 366 nm fluorescence lamp

Black Light Blue, 8 W, λ = 366 nm

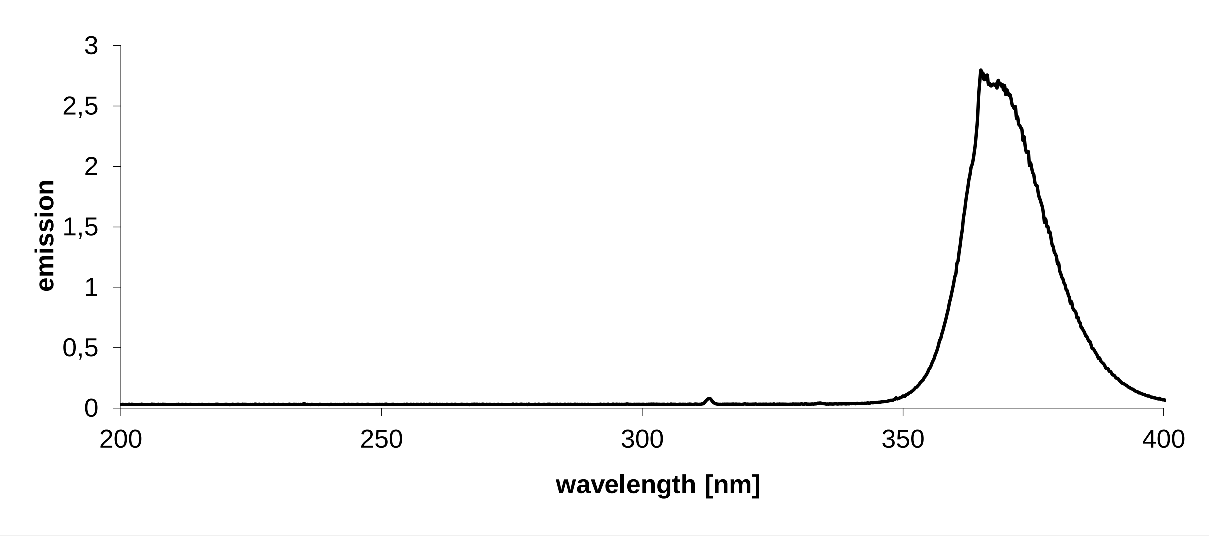

9. References

[1] C. Brenninger, A. Pöthig, T. Bach, *Angew.Chem. Int. Ed.* **2017**, *56*, 4337 – 4341; *Angew. Chem.* **2017**, *129*, 4401 – 4405.

[2] B. N. Hemric, K. Shen, Q, Wang, *J. Am. Chem. Soc.* **2016**, *138,* 5813-5816.

[3] R. Brimioulle, T. Bach, *Angew. Chem. Int. Ed.* **2014**, *53*, 12921-12924; *Angew. Chem.* **2014**, *47*, 13135-13138.

[4] P. Kraft, A. Bruneau, *Eur. J. Org. Chem.* **2007**, 2257-2267.

[5] S. Ozaki, T. Nakanishi, M. Sugiyama, C. Miyamoto, H. Ohmori, *Chem. Pharm. Bull.* **1991**, *39*, 31-35.
